# Supplementary material for: Study protocol for a population-based observational surveillance study of culture-confirmed neonatal bloodstream infections and meningitis in South Africa: Baby GERMS-SA
Source: BMJ Open. 2022 Feb 8;12(2):e049070. doi: 10.1136/bmjopen-2021-049070 (PMC8830263; doi:10.1136/bmjopen-2021-049070)
Supplement: Supplementary data [file bmjopen-2021-049070supp001.pdf]

A study protocol for a population-based observational surveillance study describing culture–confirmed neonatal bloodstream infections and meningitis in South Africa: Baby GERMS-SA

Authors

Susan Meiring[1,2]; Rudzani Mathebula[3]; Rindidzani Magobo[3]; Olga Perovic[3,4]; Vanessa Quan[1]; Cheryl Cohen[2,5]; Linda de Gouveia[5]; Anne von Gottberg[4,5,6]; Cheryl Mackay[7]; Mphekwa T. Mailula[8]; Rose Phayane[9]; Angela Dramowski[10]; Nelesh P. Govender[3,4,6] for Baby GERMS-SA

Affiliations

1. Division of Public Health Surveillance and Response, National Institute for Communicable Diseases, a Division of the National Health Laboratory Service, Johannesburg, South Africa
2. School of Public Health, Faculty of Health Sciences, University of the Witwatersrand, Johannesburg, South Africa
3. Centre for Healthcare-Associated Infections, Antimicrobial Resistance and Mycoses, National Institute for Communicable Diseases, a Division of the National Health Laboratory Service, Johannesburg, South Africa
4. Department of Clinical Microbiology and Infectious Diseases, School of Pathology, Faculty of Health Sciences, University of Witwatersrand, Johannesburg, South Africa
5. Centre for Respiratory Diseases and Meningitis, National Institute for Communicable Diseases, a Division of the National Health Laboratory Service, Johannesburg, South Africa
6. Division of Medical Microbiology, School of Pathology, Faculty of Health Sciences, University of Cape Town, South Africa
7. Department of Paediatrics and Child Health, Dora Nginza Hospital, Nelson Mandela Bay, South Africa
8. Department of Paediatrics and Child Health, Mankweng Regional Hospital, Mankweng, South Africa
9. Department of Paediatrics and Child Health, Tembisa Provincial Hospital, Johannesburg, South Africa
10. Department of Paediatrics and Child Health, Division of Paediatric Infectious Diseases, Faculty of Medicine and Health Sciences, Stellenbosch University, Cape Town, South Africa

Meiring S, *et al. BMJ Open* 2022; 12:e049070. doi: 10.1136/bmjopen-2021-049070

Supplementary table: Baby GERMS neonatal sepsis surveillance project data dictionary for tier 2 enhanced surveillance data collection tool on REDCap

Variable / Field Name	Form Name	Field Type	Field Label
study_id	lab_form	text	Patient Study ID
episode_no	lab_form	text	Episode Number
patient_surname	lab_form	text	Patient Surname
patient_name	lab_form	text	Patient First Names
birth_date	lab_form	text	Date of birth
gender	lab_form	text	Gender
folder_no	lab_form	text	Folder Number
spec_date	lab_form	text	First specimen collection date
hosp_name	lab_form	text	Hospital name
province_name	lab_form	text	Province name
spec_type	lab_form	text	Specimen type
organism_name	lab_form	text	Organism Name
received_by_charm	lab_form	text	Received by CHARM
specm_date	isolates	text	Specimen Collection date
nicu_admdate	treatment_history	text	NICU admission date
pt_ref	treatment_history	dropdown	Was patient referred from another hospital
ref_date	treatment_history	text	Date of admission at [ref_facility]
ref_facility	treatment_history	text	If Yes, state facility name where patient was referred from
ref_days	treatment_history	calc	Number of days at referred facility [ref_facility] before transferred to [hosp_name]
adm_birth_yes	treatment_history	radio	Was the baby admitted since birth [yes/no]
trans_outfac	treatment_history	dropdown	Was patient transferred to another hospital?
date_transf	treatment_history	text	Date transferred out to [fac_name]
fac_name	treatment_history	text	If Yes, specify hospital name
momhiv_status	treatment_history	radio	Maternal HIV Status of [mom_surname] [mom_name]
hivstatus_child	treatment_history	radio	HIV status child
hivtest	treatment_history	radio	HIV test type
hiv_date	treatment_history	text	HIV Test date
child_exposure	treatment_history	radio	Was the child exposed to HIV-infection [exposed/unexposed]

antib_prior	treatment_history	dropdown	Antimicrobial treatment in 2 weeks prior to culture?
diagn_organism	treatment_history	checkbox	Diagnosis related to organism isolated
del_mode	antenatal_history	dropdown	Type of delivery
gestage	antenatal_history	text	Gestational age at birth
prem_reason	antenatal_history	radio	If Gestational age of [gestage] weeks is below 37, state premature reasons
mat_cond	antenatal_history	checkbox	Maternal condition
fet_cond	antenatal_history	checkbox	Fetal conditions
antib_intrap	antenatal_history	dropdown	Were intrapartum antibiotics given to the mother?
antib_intra	antenatal_history	checkbox	If YES state antibiotics prescribed and dates
birth_wght	antenatal_history	text	Birth weight
pregn_type	antenatal_history	dropdown	Pregnancy type [singleton, twin, triplet, etc]
birth_plc	antenatal_history	dropdown	Place of birth
home_birth	antenatal_history	dropdown	If delivery at Home, was birth attended?
resusc	antenatal_history	dropdown	Was resuscitation required at birth?
resusc_type	antenatal_history	radio	Types of resuscitation
resusc_oth	antenatal_history	text	If Other resuscitation, specify
apgr_1min	antenatal_history	text	Apgar score at 1 minute
apgr_5min	antenatal_history	text	Apgar score at 5 minutes
apgr_10min	antenatal_history	text	Apgar score at 10 minutes
anten_steroids	antenatal_history	dropdown	Antenatal steroids use
preg_num	antenatal_history	text	Total number of pregnancies (gravidity)
preg_birth	antenatal_history	text	Total number of births (parity) include this child in the number
mom_age	antenatal_history	text	Maternal age of [mom_surname] [mom_name] in Years
mom_educ	antenatal_history	dropdown	Maternal education of [mom_surname] [mom_name]
relat_status	antenatal_history	dropdown	Relationship status of [mom_surname] [mom_name]
empl_status	antenatal_history	dropdown	Employment status of Mother, [mom_name] [mom_surname]
preg_compl	antenatal_history	radio	Any pregnancy related infection?

curr_wght	medical_history	text	Current weight done closest to specimen date [spec_date]
invasive	medical_history	radio	Invasive devices required by the child?
invas_spec	medical_history	checkbox	If Invasive = [invasive] specify, select all that apply
pulse_rate	medical_history	radio	Pulse rate
resp_rate	medical_history	text	Respiratory rate
tacchyp_yes	medical_history	radio	Tacchypnea present?
tacchyp_no	medical_history	radio	Tacchypnea present?
oxyg_res	medical_history	text	Oxygen saturation
bld_syst	medical_history	text	Blood pressure result (Systolic)
bld_diast	medical_history	text	Blood pressure result (Diastolic)
temp_rest	medical_history	text	Temperature result
resp_dist	medical_history	calc	Respiratory distress symptoms?, (Yes, if any of options 1-7 is checked
resp_req	medical_history	calc	Respiratory support required? (Yes, if any of options 1-6 is checked, No, if None is checked)
resp_improv	medical_history	radio	Has respiratory support increased since day prior to specimen collection? Ie increased ventilation, new intubation, more O2 required
feed_prob	medical_history	calc	Does the infant have feeding problems
cardio_inst	medical_history	calc	Cardio vascular instability indicated?
cns_symp	medical_history	checkbox	Central Nervous System symptoms?
umbil	medical_history	radio	Umplical sepsis noted?
h2_startdate	medical_history	text	H2 blockers started?
curr_feed	medical_history	checkbox	Current feeding
feed_type	medical_history	checkbox	Type of feeding
abnorm_oth	medical_history	text	Other underlying abnormality, specify
antib_pos	medical_history	dropdown	Antimicrobial treatment at time of positive culture?
wcc_count	medical_history	text	White cell count (WCC) results
platelet_count	medical_history	text	Platelets
abg_res	medical_history	text	Arterial Blood Gas STANDARD BASE EXCESS (SBE in mmol/L or mEq/L)

ph_result	medical_history	text	What was the patients pH?
pco2_result	medical_history	text	What was the patients pCO2
co2_unit	medical_history	radio	What units is the pCO2 measured in
hc03_result	medical_history	text	What was the patients HCO3 (in mmol/L)
lumbar	medical_history	radio	Was lumbar puncture performed for positive culture?
csf_wcc	medical_history	text	CSF WCC results
csf_res	medical_history	radio	CSF Pleocytosis (for babies < 28 days and cell count >20cells/mm3; or babies >28 days cell count >10cells/mm3)
finout_inf	main_outcome	radio	Final outcome at end of hospitalisation or after 30 days of specimen collection if still admitted for other reasons
fin_date	main_outcome	text	Date of final outcome infant
neon_date	main_outcome	text	Date that NEONATE was 28 days old calculated from [birth_date] plus 27 days
outcome_neon	main_outcome	radio	Neonate Outcome at 28 days

Meiring S, *et al. BMJ Open* 2022; 12:e049070. doi: 10.1136/bmjopen-2021-049070
