# Supplementary material for: Study protocol for a population-based observational surveillance study of culture-confirmed neonatal bloodstream infections and meningitis in South Africa: Baby GERMS-SA
Source: BMJ Open. 2022 Feb 8;12(2):e049070. doi: 10.1136/bmjopen-2021-049070 (PMC8830263; doi:10.1136/bmjopen-2021-049070)
Supplement: Author's manuscript [file bmjopen-2021-049070.draft_revisions.pdf]

BMJ Open

BMJ Open is committed to open peer review. As part of this commitment we make the peer review history of every article we publish publicly available.

When an article is published we post the peer reviewers' comments and the authors' responses online. We also post the versions of the paper that were used during peer review. These are the versions that the peer review comments apply to.

The versions of the paper that follow are the versions that were submitted during the peer review process. They are not the versions of record or the final published versions. They should not be cited or distributed as the published version of this manuscript.

BMJ Open is an open access journal and the full, final, typeset and author-corrected version of record of the manuscript is available on our site with no access controls, subscription charges or pay-per-view fees (http://bmjopen.bmj.com).

If you have any questions on BMJ Open's open peer review process please email info.bmjopen@bmj.com

BMJ Open

Baby GERMS-SA: surveillance for culture-confirmed neonatal bloodstream infections and meningitis in South Africa

Journal:	*BMJ Open*
Manuscript ID	bmjopen-2021-049070
Article Type:	Protocol
Date Submitted by the Author:	17-Jan-2021
Complete List of Authors:	Meiring, Susan; National Institute for Communicable Diseases; University of the Witwatersrand Faculty of Health Sciences<br>Mathebula, Rudzani; National Institute for Communicable Diseases<br>Magobo, Rindidzani; National Institute for Communicable Diseases<br>Perovic, Olga; National Institute for Communicable Diseases; University of the Witwatersrand Faculty of Health Sciences, Department of Clinical Microbiology and Infectious Diseases<br>Quan, Vanessa; National Institute for Communicable Diseases<br>Cohen, Cheryl; National Institute for Communicable Diseases<br>de Gouveia, Linda; National Institute for Communicable Diseases<br>von Gottberg, Anne ; National Institute for Communicable Diseases; University of the Witwatersrand Faculty of Health Sciences<br>Mackay, Cheryl; Dora Nginza Hospital, Department of Paediatrics and Child Health<br>Mailula, Mphekwa; Mankweng Regional Hospital Mankweng, Department of Paediatrics and Child Health<br>Phayane, Rose; Tembisa Provincial Hospital, Department of Paediatrics and Child Health<br>Dramowski, Angela; Stellenbosch University, Department of Paediatrics and Child Health, Division of Paediatric Infectious Diseases, Faculty of Medicine and Health Sciences<br>Govender, Nelesh; National Institute for Communicable Diseases; University of the Witwatersrand Faculty of Health Sciences
Keywords:	NEONATOLOGY, INFECTIOUS DISEASES, Paediatric infectious disease & immunisation < PAEDIATRICS, PUBLIC HEALTH, Microbiology < PATHOLOGY, Paediatric pathology < PAEDIATRICS

SCHOLARONE™
Manuscripts

Title

Baby GERMS-SA: surveillance for culture-confirmed neonatal bloodstream infections and meningitis in South Africa

Authors

Susan Meiring[1,2]; Rudzani Mathebula[3]; Rindidzani Magobo[3]; Olga Perovic[3,4]; Vanessa Quan[1]; Cheryl Cohen[2,5]; Linda de Gouveia[5]; Anne von Gottberg[4,5,6]; Cheryl Mackay[7]; Mphekwa T. Mailula[8]; Rose Phayane[9]; Angela Dramowski[10]; Nelesh P. Govender[3,4,6] for Baby GERMS-SA

Affiliations

1. Division of Public Health Surveillance and Response, National Institute for Communicable Diseases, a Division of the National Health Laboratory Service, Johannesburg, South Africa
2. School of Public Health, Faculty of Health Sciences, University of the Witwatersrand, Johannesburg, South Africa
3. Centre for Healthcare-Associated Infections, Antimicrobial Resistance and Mycoses, National Institute for Communicable Diseases, a Division of the National Health Laboratory Service, Johannesburg, South Africa
4. Department of Clinical Microbiology and Infectious Diseases, School of Pathology, Faculty of Health Sciences, University of Witwatersrand, Johannesburg, South Africa
5. Centre for Respiratory Diseases and Meningitis, National Institute for Communicable Diseases, a Division of the National Health Laboratory Service, Johannesburg, South Africa
6. Division of Medical Microbiology, School of Pathology, Faculty of Health Sciences, University of Cape Town, South Africa
7. Department of Paediatrics and Child Health, Dora Nginza Hospital, Nelson Mandela Bay, South Africa
8. Department of Paediatrics and Child Health, Mankweng Regional Hospital, Mankweng, South Africa
9. Department of Paediatrics and Child Health, Tembisa Provincial Hospital, Johannesburg, South Africa

For peer review only

For peer review only - http://bmjopen.bmj.com/site/about/guidelines.xhtml

10. Department of Paediatrics and Child Health, Division of Paediatric Infectious Diseases, Faculty of Medicine and Health Sciences, Stellenbosch University, Cape Town, South Africa

Corresponding author

Dr Susan Meiring

Address: C18 Microbiology Department, NHLS, Groote Schuur Hospital, Anzio Road, Observatory, 7925, South Africa.

Telephone: +27 21 404 5540 +27 84 597 3688

Email: susan.meiring@nhls.ac.za

Key words

Neonates; sepsis; bloodstream infection; meningitis; South Africa; surveillance; antimicrobial resistance

Word count:

Abstract: 249 (250)

Main body: 3272

Abstract

Introduction:

Worldwide, neonatal mortality remains high accounting for 47% of childhood deaths in 2019 and including an estimated 500 000 deaths from neonatal infections. While 42% of global neonatal deaths occur in Sub-Saharan Africa, there is limited understanding of population-level burden and aetiology of neonatal infections outside tertiary-level institutions.

Methods:

We aim to implement the first population-level surveillance for bloodstream infections and meningitis among neonates aged <28 days in South Africa. Tier 1 will include national surveillance of culture-confirmed neonatal infections at all public-sector hospitals describing

For peer review only

For peer review only - http://bmjopen.bmj.com/site/about/guidelines.xhtml

infection incidence risk, pathogen profile and antimicrobial susceptibility by institution, province and healthcare level (2014-2021). Tier 2 (nested within tier 1) will be conducted at 6 regional neonatal units over 12 months, will compare the clinical characteristics of neonates with early- and late-onset infections and identify potentially-modifiable risk factors for mortality. Through tier 2, we will determine the antimicrobial susceptibility of neonatal pathogens, evaluate the appropriateness of empiric antibiotic prescribing and determine the genomic epidemiology of multi-drug resistant bacterial and fungal pathogens.

Discussion:

Baby GERMS-SA aims to impact on national policy, resource allocation and guidelines by describing the national burden of neonatal infections in South Africa (tier 1) and a detailed characterisation of risk factors, outcomes and antimicrobial-resistant pathogens associated with bacterial and fungal infections at sentinel units (tier 2). This will be achieved by setting up a sustainable and in-country-led surveillance system that will be used to monitor the impact of future public health interventions aimed at reducing infection-related mortality in neonates.

Summary: Strengths and Limitations

- Baby GERMS-SA will be the first population-level surveillance study to determine the aetiology of culture-confirmed neonatal infections in an African country.
- This study will provide baseline incidence estimates for culture-confirmed early- and late-onset infections in neonatal units at all levels of health care in South Africa. In addition, the study will determine the frequency of neonatal infection clusters and outbreaks.
- Enhanced surveillance at sentinel regional neonatal units (Level 2) will establish the antimicrobial susceptibility profile of neonatal pathogens and evaluate the appropriateness of empiric antibiotic prescribing for sepsis in these units.
- Since laboratory-based surveillance relies heavily on adequate specimen collection and laboratory diagnostic capacity, Baby GERMS-SA may underestimate neonatal infection burden in rural districts of South Africa.

For peer review only - http://bmjopen.bmj.com/site/about/guidelines.xhtml

- The Baby GERMS-SA surveillance study will describe the burden of neonatal infections in South Africa and identify modifiable risk factors which could be targeted to reduce neonatal morbidity and mortality.

Introduction

Worldwide, neonatal mortality remains high, despite a substantial decline in under-5 childhood deaths from 12.7 million in 1990 to 5.2 million in 2019.[1][2] Neonatal deaths accounted for 47% of all under-5 childhood deaths in 2019, with infectious causes being the third highest contributors to neonatal mortality, following prematurity and intra-partum related events.[2] Infectious diseases caused approximately 500 000 neonatal and 1.5 million under-5 childhood deaths in 2017.[3][2]

While 42% of global neonatal deaths occur in Sub-Saharan Africa, the population-level burden and aetiology of neonatal infections is not well understood.[1,4,5] Studies in Africa have been limited to tertiary-level institutions, with no population-based surveillance studies reporting on neonatal infection incidence risks or rates.[5][6–8] Contributing factors to this lack of data include under-utilization or unavailability of hospital-based services for neonatal care, suboptimal specimen collection to confirm an infectious disease diagnosis, limited capacity of diagnostic pathology laboratories to detect, identify and characterize neonatal pathogens, absence of appropriate denominator data for calculating incidence risks or rates, lack of clinical data to differentiate between infection types (i.e. healthcare-associated infections versus vertical transmission of pathogens causing early-onset sepsis), and limited resources for setting up and maintaining population-based surveillance studies.[9–11]

The South African government seeks to reduce neonatal sepsis rates by 84% nationally by 2025 through various strategies across the continuum of maternal and newborn care.[12][13] However, unless the national burden of laboratory-confirmed neonatal infections occurring at all levels of health-care in South Africa can be clearly documented, measuring the effectiveness of these interventions against a baseline will be difficult.

We aim to improve the reporting of neonatal infection burden and determine the risk factors for mortality associated with neonatal infections in urban and rural South Africa

For peer review only - http://bmjopen.bmj.com/site/about/guidelines.xhtml

using a two-tiered surveillance study. We will describe the incidence of culture-confirmed neonatal bloodstream infections and meningitis by province, pathogen-specific aetiology and antimicrobial susceptibility at different levels of health-care over an eight-year period in tier 1. We will describe the clinical characteristics of culture-confirmed cases, identify modifiable risk factors associated with mortality and describe the antimicrobial susceptibility and genomic epidemiology of multi-drug resistant bacterial and fungal pathogens over 12 months in tier 2.

Methods

Hypothesis:

We hypothesise that the incidence risk of culture-confirmed neonatal infections has increased over the study period in South Africa, owing to in-hospital transmission of multi-drug-resistant organisms. In addition, we hypothesise that neonatal deaths due to infections may be related to modifiable risk factors such as low rates of antenatal steroid use in preterm infants, low rates of breastfeeding amongst neonates who develop infections, and prolonged use of indwelling catheters.[14,15]

Study objectives

Baby GERMS-SA has three main objectives:

i. To determine the bacterial and fungal aetiology and incidence risk of culture-confirmed infections amongst neonates presenting to all levels of hospital-based care in South Africa from 2014 to 2021

ii. To confirm the bacterial and fungal aetiology and prevalence of antimicrobial resistance in pathogens causing neonatal infections at secondary-level health-care facilities over a 12-month period in South Africa.

iii. To determine the characteristics of neonates who are diagnosed with culture-confirmed infections at secondary-level health care facilities and identify potentially-modifiable risk factors for death

For peer review only - http://bmjopen.bmj.com/site/about/guidelines.xhtml
For peer review only

Study design

Two complementary surveillance approaches will be used in this study. First, retrospective population-based surveillance will be established to identify culture-confirmed episodes of neonatal infections occurring in all public health facilities in South Africa from 2014 through to 2021, and population denominator data on live births will be used to calculate national and provincial incidence risks of infection (Tier 1). Second, prospective enhanced laboratory-based surveillance will be conducted at six sentinel neonatal units to collect detailed clinical data from neonates with infection and to determine risk factors for mortality (Tier 2). The overall and pathogen-specific incidence rate of neonatal infections will be calculated at sentinel sites using patient bed-days as a denominator. All-cause mortality rates will also be calculated. The sentinel neonatal units will be selected from a list of secondary level/ regional public-sector hospitals with inpatient neonatal services. Only one institution will be selected per province. In addition, a cross-sectional electronic survey will be conducted at a sample of large public-sector neonatal units in South Africa to determine available bed and staff resources, understand infection prevention and antimicrobial stewardship practices and obtain admission denominator data.

Definitions

A neonate will be defined as a child aged <28 days with further categorisation into early (0-6 days) and late neonatal periods (7-27 days). The post-neonatal period will be defined as the period from 28-60 days.

We will use a laboratory-based case definition for neonatal invasive infections based on Level 1 of the Brighton Collaboration Neonatal Infections Working Group for neonatal invasive bloodstream infections.[16] This includes any neonate/infant who is admitted to a public-sector hospital with a recognised pathogen (bacteria or fungi) identified using a validated method from a normally-sterile site (blood or cerebrospinal fluid [CSF]) or a normally non-pathogenic organism, e.g. coagulase-negative staphylococci isolated from 2 invasive specimen cultures taken at 2 different time points within 14-days. We will use a 14-day period from the date of the first positive culture to define an episode of infection. This case definition makes the assumptions that: i) the neonate/ infant would not have been evaluated for sepsis (i.e. had specimens collected for culture) in the absence of clinical signs

For peer review only - http://bmjopen.bmj.com/site/about/guidelines.xhtml

or a clear clinical indication and ii) the isolated bacteria/ fungi are not contaminants and iii) that most neonatal bacterial or fungal infections can be cleared within 14 days with appropriate antimicrobial treatment. We will use a 72-hour age cut off to distinguish early-onset (less than 72 hours since birth) from healthcare-associated ($\geq$ 72 hours since birth) neonatal infections.

Study processes, Training and Analyses

Tier 1: National population-based laboratory-based surveillance

Positive blood and CSF microbiology culture results from patients admitted to public health institutions in South Africa will be obtained from a surveillance data warehouse which archives data from TrakCare, the electronic National Health Laboratory Service (NHLS) laboratory information system in use since at least 2014. We will request data on positive blood and CSF cultures among infants aged <12 months for at least an 8-year period (from 1 January 2014 through to 31 December 2021). Based on a preliminary analysis of 2016 data, we estimate 7 000-8 000 laboratory-confirmed neonatal infections to be reported each year from approximately 180 public-sector hospitals. (Figure 1) The following variables will be requested: laboratory name, province, district, sub-district, hospital name, ward name/ type, patient first name and surname, laboratory episode number, data warehouse unique identifier, patient date of birth, date of specimen collection, specimen type, microscopy (including Gram stain and CSF cell counts) and culture result, identification of pathogen and antimicrobial susceptibility results. Patient identifying information (i.e. name, date of birth) will be requested in order to accurately de-duplicate records; this is also the current practice in the "parent" GERMS-SA surveillance programme.[17] This information is essential to distinguish neonatal and maternal specimens (in the first few days of life, clinicians often send specimens labelled with the mother's details). Neonatal date of birth is also an essential piece of information to determine timing of infection (early versus late onset). A national surveillance dataset will be created containing de-duplicated laboratory records of neonates with laboratory-confirmed bloodstream and CSF infections. These data will be cleaned and analysed using Stata version 15 (StataCorp Inc., College Station, Texas, USA).

Data from the first tier of national laboratory-based surveillance will be used to calculate the incidence risk of neonatal sepsis stratified by level of healthcare (district (level 1), regional

For peer review only - http://bmjopen.bmj.com/site/about/guidelines.xhtml

(level 2) or tertiary/referral (level 3)), geographic region (province, district, sub-district) and timing of infection. We will use national neonatal unit admissions (if available through the cross-sectional survey) or live births in the total population as denominators for incidence risk calculations.[18] The main analysis (incidence risk calculations) may focus on provinces where specimen-collection practices are more consistent and estimates of incidence risks are more likely to be valid. Missing data will be imputed for the stratified incidence risk calculations. In line with NICD's mandate, we will endeavour to make aggregate data publicly available through a neonatal infection dashboard displaying interactive maps and graphs to district level (similar to the antimicrobial resistance dashboard available at www.nicd.ac.za).

Tier 2: Enhanced sentinel site laboratory-confirmed neonatal sepsis surveillance

The six sentinel regional hospitals and their provinces include: Dora Nginza Provincial Hospital (Eastern Cape Province), Tembisa Hospital (Gauteng Province), Mankweng Hospital (Limpopo Province), Klerksdorp (North West Province), Queen Nandi Regional Hospital (KwaZulu-Natal Province) and Rob Ferreira Hospital (Mpumalanga Province) (Figure 1). In 2016, thirteen per cent (901/7 124) of all culture-confirmed neonatal infection episodes in South Africa occurred in neonates admitted to these facilities. (Figure 1) NHLS microbiology laboratories serving each of the six sentinel surveillance sites will be requested to prospectively submit any cultured bloodstream or CSF isolate from infants aged <12 months on Dorset transport media (Media Mage, Johannesburg) to the NICD reference laboratories for further characterisation. NICD personnel will provide training to participating laboratories to ensure that neonatal specimens are optimally processed and quality control measures are adhered to. In addition, training on diagnosis of neonatal sepsis and meningitis will be provided to clinicians. Data on basic demographic details of the infants and NHLS laboratory characterisation of the isolates will be transferred from the data warehouse directly into REDCap, an electronic data capture tool hosted at the University of the Witwatersrand, and reference laboratory isolate characterisation and clinical data from the infants will be added to this dataset. [19,20] We will prospectively monitor cases of neonatal infections at the sentinel hospitals and upon request, conduct investigations for any potential clusters/ outbreaks. These activities are covered by a separate ethics application (WITS HREC reference: M160667 – Essential communicable diseases surveillance

For peer review only - http://bmjopen.bmj.com/site/about/guidelines.xhtml
For peer review only

and outbreak investigation activities of the NICD). For each episode of culture-confirmed infection, we will perform a retrospective medical record review using a standardised data abstraction tool (supplementary table). Inpatient medical charts will be scanned after patient identifiers are physically blocked by a card with a unique study identifier. These imaged records will be abstracted electronically off-site by trained medical and nursing study personnel into a REDCap database.

A dataset will be generated containing all cases of neonatal infection from the six sentinel hospitals. This will include demographic and baseline clinical details of enrolled participants, clinical updates during their hospital admission, antimicrobial therapy during the admission, in-hospital outcome and detailed laboratory characterisation of the isolate/s causing infection. The following variables will be collected: i) Isolate data: laboratory name, province, district, sub-district, hospital name, ward name/ type, patient name and surname, laboratory episode number, data warehouse unique identifier, date of birth, date of admission, date of specimen collection, specimen type, microscopy and culture result, antimicrobial susceptibility results, NICD reference laboratory data including whole genome sequencing (WGS) where applicable. Additional patient data: basic demographic data, type of admitting unit, date of birth, gestational age at birth, mode of delivery, 5-min Apgar score, date of admission, comorbid neonatal conditions, maternal and neonatal risk factors for infection, maternal HIV status (and baby's HIV-PCR result if tested), presence of central venous lines, respiratory support (non-invasive, invasive, surfactant administration), clinical presentation on day of specimen collection, inflammatory markers of infection on day of specimen collection, empiric and directed antibiotic/antifungal therapy (determine if "appropriate" based on organism susceptibility), complications of infection, in-hospital outcome at 28 days (for neonates) or at the end of admission (for neonates and infants who are admitted for longer periods).

The following analyses will be performed using this dataset: i) A description of the characteristics of neonates with culture-confirmed bloodstream infection and meningitis by pathogen; ii) A description of the antimicrobial susceptibility of the most important bacterial and fungal pathogens causing neonatal infections in South Africa over time and appropriateness of current empiric regimens for early and late-onset infections iii) The incidence risk of infection by pathogen per 1 000 live births in the catchment area or per 1

For peer review only - http://bmjopen.bmj.com/site/about/guidelines.xhtml

000 inpatient days will be calculated for neonates (0-27 days) iv) An estimation of the outcomes following neonatal infection and the risk factors associated with death v) Multivariable analyses will be performed to determine potentially-modifiable risk factors for mortality for various subgroups (e.g. effect of receiving antenatal steroids on mortality amongst preterm infants; effect of feeding mode on mortality; effect of prolonged use of indwelling catheters on mortality among those with late-onset healthcare-associated infections). For each analysis, we will adjust for potential confounders (such as sex, birthweight, preterm birth) as appropriate.

As this dataset will contain confidential information whereby individuals could potentially be identified, the complete dataset will not be available publically. However, should external researchers request any of this information, completely de-identified data may be released following signing of a data-sharing agreement between the NICD and the requestor.

<u>Electronic survey of neonatal units with previously-identified episodes of neonatal sepsis</u>

A baseline survey of neonatal units will be conducted in 2020 to improve our understanding of the current functioning of neonatal units at various health-care levels in South Africa. A standardised questionnaire will be sent to all dedicated in-patient neonatal units for the facility manager to complete in hard copy, electronically or online using the SurveyMonkey application. Of 7 124 episodes of laboratory-confirmed neonatal infections diagnosed at 179 South African public hospitals in 2016, 97% (6 919 episodes) were from 90 hospitals that had >5 episodes of infection. We will approach these 90 hospitals for the neonatal unit survey. (Figure 1) Variables to be collected will include: hospital location, bed census in the neonatal unit, number of neonatal unit staff members, on-site or off-site laboratory services, infection prevention and control (IPC) and clinical microbiologist support for the unit and detailed statistics on number of patient-days per month and deaths per month from the neonatal unit in 2019/2020. Clinical criteria used for suspicion of sepsis or bloodstream infections, criteria for obtaining blood/CSF specimens for culture from neonates and institutional antibiotic guidance for early- and late-onset sepsis will be also be collected. Completed questionnaire data will be captured into Microsoft Excel and analysis performed using Stata. We will use denominator data obtained through the survey (e.g. admissions, patient-days) for incidence risk/ rate calculations at tier 2 facilities.

For peer review only - http://bmjopen.bmj.com/site/about/guidelines.xhtml

Data dissemination

The information gained through the surveillance study will be shared with the South African Department of Health, and various other in-country and international stakeholders. Internally, the data will be presented to the National Advisory Group on Immunisations, the Ministerial Advisory Committee on Antimicrobial Resistance, Neonatal Sepsis Task Force and the multi-sectoral National Outbreak Response Team, as well as at local and international conferences.[21] We plan to publish the results in a policy-briefing and in relevant peer-reviewed medical journals. A facility-level dashboard used to display key indicators based on the surveillance data will be set up and made available to end-users in neonatal units.

Beneficiaries

We will gain a better understanding of the burden of neonatal infections and the antimicrobial susceptibility and molecular relatedness of neonatal bacterial and fungal pathogens in an upper middle-income country, particularly in secondary-level institutions serving peri-urban and rural communities. The National Department of Health could use these data to design appropriate interventions such as antimicrobial stewardship and infection prevention and control programmes, to prioritise facilities requiring urgent intervention and to tailor these interventions for those at highest risk of neonatal sepsis. The hospitals at which we will conduct enhanced surveillance will benefit from the additional information that will come from further characterization of the isolates causing neonatal infections and gain an understanding on how they can tailor their empiric antimicrobial regimens to fit the spectrum of organisms that are being cultured. Individual neonates at these hospitals will thus benefit from the doctors adjusting their empirical therapy accordingly. We will strongly encourage enhanced surveillance sites to implement local antimicrobial stewardship and infection prevention and control programs for neonatal units and will design facility-level reports based on their local surveillance data to allow them to monitor key indicators for neonatal sepsis. Policy makers can use the data on burden of disease, mortality and risk factors associated with neonatal sepsis and the aetiological patterns of pathogens causing neonatal sepsis to align their strategies on the Continuum of Maternal and Newborn Care to help meet South Africa's goal to reduce neonatal sepsis by 84% by the year 2025. We hope that by setting up this surveillance study,

For peer review only - http://bmjopen.bmj.com/site/about/guidelines.xhtml

we will facilitate future sustainable funding of the project and will be able to objectively record the change in incidence risk of neonatal infections over time as new interventions are implemented. Ultimately, we hope that policies put in place through the data generated by this project will save the lives of many newborn babies and improve the quality of life of others in the years ahead.

Ethics and funding

The study has been approved by the Human Research Ethics Committee of the University of the Witwatersrand (M190320). Approvals for the tier 2 surveillance study was received from each provincial research committee through registration on the National Health Research Database. Funding for the study was awarded as a grant to N.P.G. from the Bill and Melinda Gates Foundation (OPP1208882).

Discussion

Unless baseline data on neonatal infections are reliably and systematically collected and analysed, there will be no objective record against which interventions aimed at reducing burden of disease in this vulnerable population can be measured. The Baby GERMS-SA national surveillance system will provide a robust platform to determine national incidence risk of neonatal infection by pathogen, level of hospital care and geographic region. This surveillance study will also be used to assess trends in the incidence risk of neonatal infections over time, thus providing an objective record by which to measure the impact of any intervention implemented in future.

The enhanced surveillance tier will focus on neonatal sepsis and meningitis occurring at sentinel hospitals. This surveillance tier will provide insight into the similarities/differences in pathogens and their antimicrobial susceptibility patterns causing neonatal sepsis by level of health care, and risk factors for mortality among neonates admitted to regional hospitals. The NICD has experience in conducting surveillance for laboratory-confirmed meningitis as well as active surveillance of several invasive bacterial and fungal diseases at a national level.[22,23] We have previously published data indicating how public health interventions such as new vaccines, antiretroviral treatment, cryptococcal antigen screening and treatment have reduced the burden of disease/ mortality caused by *Streptococcus*

For peer review only - http://bmjopen.bmj.com/site/about/guidelines.xhtml

pneumoniae, Haemophilus influenzae and *Cryptococcus neoformans* in South Africa.[24,25][26] We realise the value of having robust data prior to implementation of public health interventions in reporting the effectiveness of these interventions over time. We also conduct surveillance on selected healthcare-associated bloodstream infections and understand the variation of antimicrobial resistance and pathogen profiles by healthcare level and province.[27] We are aware that the study findings rely on adequate specimen collection and laboratory diagnostic capacity of each neonatal unit. Specimen taking practices might differ between units and therefore the surveillance may underestimate neonatal infection burden, especially in rural districts of South Africa. Tier one's comprehensive dataset should be able to provide an expected culture-positivity rate to describe the extent of this phenomenon. A major strength of our project is that we will gather complete data on laboratory-confirmed bloodstream infections and meningitis from the entire public-sector population and in-depth data from selected secondary-level sites in six provinces.

Ultimately, these surveillance data can be used to address Sustainable Development Goal 3 by aiming to improve neonatal and child health by using a two-tiered laboratory-based surveillance program to gain a deeper understanding of the aetiology and burden of neonatal sepsis, with a future aim of addressing these factors and thus reducing neonatal morbidity and mortality in low- and middle-income settings.

Acknowledgements

The authors would like to acknowledge the following clinical and laboratory staff involved in the protocol design, facilitation and collection of data for the Baby GERMS-SA project: Dora Nginza Hospital: Phunyezwa Mzayiya (laboratory), Shareef Abrahams (pathologist), Vanessa Pearce (laboratory), Zikhona Gabazana (research assistant (RA)), Melissa Ngubane (RA), Badikazi Matiwana (RA); Klerksdorp Hospital: Omphile Mekgoe (clinician), Sebabatso Khantsi (laboratory), Bernard Motsetse (RA), LouisaPhalatse (RA); Mankweng Hospital: Ruth Lekalakala (pathologist), Tebogo Modiba (RA), Molly Morapeli (RA); National Institute for Communicable Diseases: Linda Erasmus (pathologist), Danie Erwee (clinician), Juliet Paxton (clinician), Siyanda Dlamini (laboratory), Marshagne Smith (laboratory), Ruth Mpembe

For peer review only - http://bmjopen.bmj.com/site/about/guidelines.xhtml

(laboratory), Ntombi Dube (administrator), Relebohile Ramatsa (RA), Thembekile Zwane (masters student), Sibongile Walaza (medical epidemiologist), Erika van Schalkwyk (medical epidemiologist); Queen Nandi Hospital: ; Constance Kapongo (clinician), Meluleki Mthimkhulu (laboratory), Sandra Maphumulo (pathologist), Dianette Pearce (RA); Rob Ferreira Hospital: Lerato Motjale (clinician), Thulisile Maphosa (clinician), Greta Hoyland (laboratory), Sindile Ntuli (pathologist), Lesley Ingle (RA); Tembisa Hospital: Harishia Naidoo (clinician), Ramatlhwa Kekana (laboratory), Dina Pombo (laboratory).

The authors would like to acknowledge Jimmy Khosa from the NICD for the production of the ARCGIS map for figure 1.

Patient and public involvement

The study design and protocol was discussed with numerous paediatricians and neonatologists at various institutions across South Africa, as well as at the launch of the Neonatal Sepsis Task Force at the United SA Neonatal Association (USANA) conference in Port Elizabeth, SA, on 13 September 2019.

Funding

Funding for the study was awarded as a grant to N.P.G. from the Bill and Melinda Gates Foundation (OPP1208882).

Conflict of interest

The authors have no conflicts of interest to declare.

Author statement

The authors contributed in the following ways:

- Substantial contribution to conception and design of the study: all authors
- Revising the manuscript for important intellectual content: SM, RM, NPG, AD, VQ, OP
- Final approval of manuscript: all authors

For peer review only

For peer review only - http://bmjopen.bmj.com/site/about/guidelines.xhtml

Data statement

A facility-level dashboard used to display key indicators based on the surveillance data will be set up and made available to end-users at neonatal units. As the tier 2 dataset will contain confidential information whereby individuals could potentially be identified, the clinical dataset will not be available publically. However, should other researchers request any of this information, completely de-identified data may be released following signing of a data-sharing agreement between the NICD and the requestor.

For peer review only

Figures

Figure 1: Map of South Africa showing relative numbers of laboratory-confirmed bacterial and fungal infectious episodes amongst neonates diagnosed at each hospital site, 2016 (n=7 124)

*the six tier 2 sentinel surveillance sites are indicated in green

References

1. Sharrow D, Hug L, Liu Y, You D, United Nations Inter-agency Group for Child Mortality Estimation UNIG for CME. UN IGME Child mortality report 2019. 2019. Available at: https://www.unicef.org/media/60561/file/UN-IGME-child-mortality-report-2019.pdf.

2. Liu L, Johnson H, Cousens S. Global, regional, and national causes of child mortality in 2000-2010: an updated systematic analysis. Lancet **2015**; 385:430–440. Available at: http://dx.doi.org/10.1016/S0140-6736(14)61698-6.

3. Dicker D, Nguyen G, Abate D, et al. Global, regional, and national age-sex-specific mortality for 282 causes of death in 195 countries and territories, 1980-2017: a systematic analysis for the Global Burden of Disease Study 2017 GBD 2017 Causes of Death Collaborators*. Lancet **2018**; 392:1736–88. Available at: https://vizhub.health.

4. Seale AC, Mwaniki M, Newton CR, Berkley JA. Maternal and early onset neonatal bacterial sepsis: burden and strategies for prevention in sub-Saharan Africa. Lancet Infect Dis **2009**; 9:428–438. Available at: https://www.sciencedirect.com/science/article/pii/S1473309909701720?via%3Dihub

For peer review only - http://bmjopen.bmj.com/site/about/guidelines.xhtml

.

5. Fleischmann-Struzek C, Goldfarb DM, Schlattmann P, Schlapbach LJ, Reinhart K, Kissoon N. The global burden of paediatric and neonatal sepsis: a systematic review. Lancet Respir Med **2018**; 6:223–230. Available at: http://dx.doi.org/10.1016/S2213-2600(18)30063-8.

6. Ogunlesi TA, Ogunfowora OB, Osinupebi O, Olanrewaju DM. Changing trends in newborn sepsis in Sagamu, Nigeria: bacterial aetiology, risk factors and antibiotic susceptibility. J Paediatr Child Heal **2011**; 47:5–11. Available at: internal-pdf://185.112.96.26/Changing trends in newborn sepsis in Sagamu.pdf.

7. Hamer DH, Darmstadt GL, Carlin JB, et al. Etiology of bacteremia in young infants in six countries. Pediatr Infect Dis J **2015**; 34:e1-8.

8. Shitaye D, Asrat D, Woldeamanuel Y, Worku B. Risk factors and etiology of neonatal sepsis in Tikur Anbessa University Hospital, Ethiopia. Ethiop Med J **2010**; 48:11–21.

9. Lawn JE, Bianchi-Jassir F, Russell NJ, et al. Group B Streptococcal Disease Worldwide for Pregnant Women, Stillbirths, and Children: Why, What, and How to Undertake Estimates? Clin Infect Dis **2017**; 65:S89–S99. Available at: https://www.ncbi.nlm.nih.gov/pubmed/29117323.

10. Organisation WHNV-WHO reference number: W 0. Managing possible serious bacterial infection in young infants when referral is not feasible: Guidelines and WHO/UNICEF recommendations for implementation. 2015; Available at: http://www.who.int/maternal_child_adolescent/documents/bacterial-infection-infants/en/.

11. Williams PCM, Isaacs D, Berkley JA. Antimicrobial resistance among children in sub-Saharan Africa. Lancet Infect Dis **2018**; 18:e33–e44. Available at: https://www.ncbi.nlm.nih.gov/pmc/articles/PMC5805911/.

12. Lawn JE, Kinney M, Blencowe H, Group The Lancet Every Newborn Study. Every Newborn: Executive summary. 2014. Available at: https://els-jbs-prod-cdn.jbs.elsevierhealth.com/pb/assets/raw/Lancet/stories/series/everynewborn_exec_summ.pdf.

For peer review only

For peer review only - http://bmjopen.bmj.com/site/about/guidelines.xhtml

13. Kerber KJ, de Graft-Johnson JE, Bhutta ZA, Okong P, Starrs A, Lawn JE. Continuum of care for maternal, newborn, and child health: from slogan to service delivery. Lancet **2007**; 370:1358–1369.

14. Mwansa-Kambafwile J, Cousens S, Hansen T, Lawn JE. Antenatal steroids in preterm labour for the prevention of neonatal deaths due to complications of preterm birth. Int J Epidemiol **2010**; 39.

15. Griffin JB, Jobe AH, Rouse D, McClure EM, Goldenberg RL, Kamath-Rayne BD. Evaluating WHO-recommended interventions for preterm birth: A mathematical model of the potential reduction of preterm mortality in sub-Saharan Africa. Glob. Heal. Sci. Pract. 2019; 7:215–227.

16. Vergnano S, Buttery J, Cailes B, et al. Neonatal infections: Case definition and guidelines for data collection, analysis, and presentation of immunisation safety data. Vaccine **2016**; 34:6038–6046. Available at: internal-pdf://215.147.49.17/Vergnano-2016-Neonatal infections_ Case defini.pdf.

17. Africa G for ER and M disease S in S. GERMS-SA Annual Report 2008. GERMS-SA Annu. Rep. 2009; Available at: http://nicd.ac.za/?page=germs-sa&id=97.

18. Statistics South Africa . Statistical Release: Recorded Live Births: South Africa 2019. 2019. Available at: https://www.statssa.gov.za/publications/P0305/P03052019.pdf.

19. Harris PA, Taylor R, Thielke R, Payne J, Gonzalez N, Conde JG. Research electronic data capture (REDCap)-A metadata-driven methodology and workflow process for providing translational research informatics support. J Biomed Inform **2009**; 42:377–381.

20. Harris PA, Taylor R, Minor BL, et al. The REDCap consortium: Building an international community of software platform partners. J Biomed Inform **2019**; 95.

21. Dramowski A, Velaphi S, Reubenson G, et al. National Neonatal Sepsis Task Force launch: Supporting infection prevention and surveillance, outbreak investigation and antimicrobial stewardship in neonatal units in South Africa. South African Med J **2020**; 110:360–363.

For peer review only

For peer review only - http://bmjopen.bmj.com/site/about/guidelines.xhtml

22. GERMS-SA. GERMS-SA Annual Report, 2018. 2019; Available at: https://www.nicd.ac.za/wp-content/uploads/2019/11/GERMS-SA-AR-2018-Final.pdf.

23. Britz E, Perovic O, von Mollendorf C, et al. The Epidemiology of Meningitis among Adults in a South African Province with a High HIV Prevalence, 2009-2012. PLoS One **2016**; 11:e0163036.

24. von Gottberg A, de Gouveia L, Madhi SA, et al. Impact of conjugate Heamophilus influenzae type b (Hib) vaccine introduction in South Africa. Bull World Heal Organ **2006**; 84:811–818. Available at: internal-pdf://77.87.76.154/Von Gottberg HIB SA paper.pdf.

25. von Gottberg A, de Gouveia L, Tempia S, et al. Effects of vaccination on invasive pneumococcal disease in South Africa. N Engl J Med **2014**; 371:1889–1899. Available at: internal-pdf://199.73.122.32/NEJM 2014 IPD vaccine effects SA.pdf internal-pdf://1339213091/NEJM 2014 IPD vaccine effects SA_supplement.PDF.

26. Larson BA, Rockers PC, Bonawitz R, et al. Screening HIV-Infected patients with low cd4 counts for cryptococcal antigenemia prior to initiation of antiretroviral therapy: Cost effectiveness of alternative screening strategies in South Africa. PLoS One **2016**; 11.

27. Perovic O, Iyaloo S, Kularatne R, et al. Prevalence and Trends of Staphylococcus aureus Bacteraemia in Hospitalized Patients in South Africa, 2010 to 2012: Laboratory-Based Surveillance Mapping of Antimicrobial Resistance and Molecular Epidemiology. PLoS One **2015**; 10:e0145429. Available at: https://www.ncbi.nlm.nih.gov/pubmed/26719975.

For peer review only

For peer review only - http://bmjopen.bmj.com/site/about/guidelines.xhtml

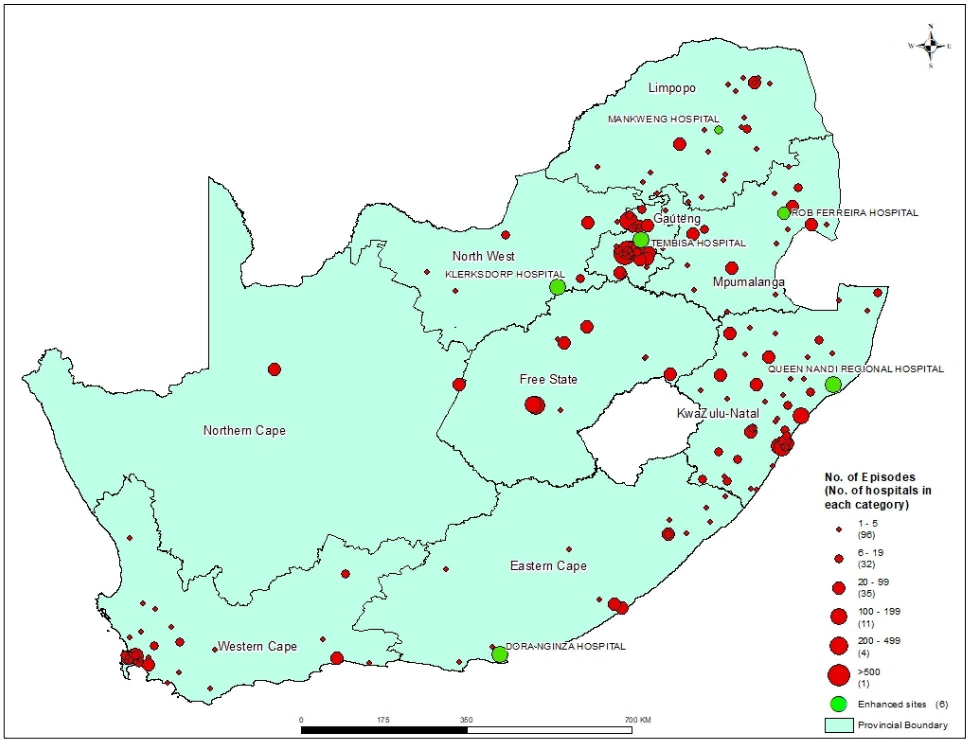

Figure 1: Map of South Africa showing relative numbers of laboratory-confirmed bacterial and fungal infectious episodes amongst neonates diagnosed at each hospital site, 2016 (n=7 124)

279x215mm (96 x 96 DPI)

Supplementary table: Baby GERMS neonatal sepsis surveillance project data dictionary for tier 2 enhanced surveillance data collection tool on REDCap

Variable / Field Name	Form Name	Field Type	Field Label
study_id	lab_form	text	Patient Study ID
episode_no	lab_form	text	Episode Number
patient_surname	lab_form	text	Patient Surname
patient_name	lab_form	text	Patient First Names
birth_date	lab_form	text	Date of birth
gender	lab_form	text	Gender
folder_no	lab_form	text	Folder Number
spec_date	lab_form	text	First specimen collection date
hosp_name	lab_form	text	Hospital name
province_name	lab_form	text	Province name
spec_type	lab_form	text	Specimen type
organism_name	lab_form	text	Organism Name
received_by_charm	lab_form	text	Received by CHARM
specm_date	isolates	text	Specimen Collection date
nicu_admdate	treatment_history	text	NICU admission date
pt_ref	treatment_history	dropdown	Was patient referred from another hospital
ref_date	treatment_history	text	Date of admission at [ref_facility]
ref_facility	treatment_history	text	If Yes, state facility name where patient was referred from
ref_days	treatment_history	calc	Number of days at referred facility [ref_facility] before transferred to [hosp_name]
adm_birth_yes	treatment_history	radio	Was the baby admitted since birth [yes/no]
trans_outfac	treatment_history	dropdown	Was patient transferred to another hospital?
date_transf	treatment_history	text	Date transferred out to [fac_name]
fac_name	treatment_history	text	If Yes, specify hospital name
momhiv_status	treatment_history	radio	Maternal HIV Status of [mom_surname] [mom_name]
hivstatus_child	treatment_history	radio	HIV status child
hivtest	treatment_history	radio	HIV test type
hiv_date	treatment_history	text	HIV Test date
child_exposure	treatment_history	radio	Was the child exposed to HIV-infection [exposed/unexposed]

For peer review only - http://bmjopen.bmj.com/site/about/guidelines.xhtml

antib_prior	treatment_history	dropdown	Antimicrobial treatment in 2 weeks prior to culture?
diagn_organism	treatment_history	checkbox	Diagnosis related to organism isolated
del_mode	antenatal_history	dropdown	Type of delivery
gestage	antenatal_history	text	Gestational age at birth
prem_reason	antenatal_history	radio	If Gestational age of [gestage] weeks is below 37, state premature reasons
mat_cond	antenatal_history	checkbox	Maternal condition
fet_cond	antenatal_history	checkbox	Fetal conditions
antib_intrap	antenatal_history	dropdown	Were intrapartum antibiotics given to the mother?
antib_intra	antenatal_history	checkbox	If YES state antibiotics prescribed and dates
birth_wght	antenatal_history	text	Birth weight
pregn_type	antenatal_history	dropdown	Pregnancy type [singleton, twin, triplet, etc]
birth_plc	antenatal_history	dropdown	Place of birth
home_birth	antenatal_history	dropdown	If delivery at Home, was birth attended?
resusc	antenatal_history	dropdown	Was resuscitation required at birth?
resusc_type	antenatal_history	radio	Types of resuscitation
resusc_oth	antenatal_history	text	If Other resuscitation, specify
apgr_1min	antenatal_history	text	Apgar score at 1 minute
apgr_5min	antenatal_history	text	Apgar score at 5 minutes
apgr_10min	antenatal_history	text	Apgar score at 10 minutes
anten_steroids	antenatal_history	dropdown	Antenatal steroids use
preg_num	antenatal_history	text	Total number of pregnancies (gravidity)
preg_birth	antenatal_history	text	Total number of births (parity) include this child in the number
mom_age	antenatal_history	text	Maternal age of [mom_surname] [mom_name] in Years
mom_educ	antenatal_history	dropdown	Maternal education of [mom_surname] [mom_name]
relat_status	antenatal_history	dropdown	Relationship status of [mom_surname] [mom_name]
empl_status	antenatal_history	dropdown	Employment status of Mother, [mom_name] [mom_surname]

For peer review only

For peer review only - http://bmjopen.bmj.com/site/about/guidelines.xhtml

preg_compl	antenatal_history	radio	Any pregnancy related infection?
curr_wght	medical_history	text	Current weight done closest to specimen date [spec_date]
invasive	medical_history	radio	Invasive devices required by the child?
invas_spec	medical_history	checkbox	If Invasive = [invasive] specify, select all that apply
pulse_rate	medical_history	radio	Pulse rate
resp_rate	medical_history	text	Respiratory rate
tacchyp_yes	medical_history	radio	Tacchypnea present?
tacchyp_no	medical_history	radio	Tacchypnea present?
oxyg_res	medical_history	text	Oxygen saturation
bld_syst	medical_history	text	Blood pressure result (Systolic)
bld_diast	medical_history	text	Blood pressure result (Diastolic)
temp_rest	medical_history	text	Temperature result
resp_dist	medical_history	calc	Respiratory distress symptoms?, (Yes, if any of options 1-7 is checked
resp_req	medical_history	calc	Respiratory support required? (Yes, if any of options 1-6 is checked, No, if None is checked)
resp_improv	medical_history	radio	Has respiratory support increased since day prior to specimen collection? Ie increased ventilation, new intubation, more O2 required
feed_prob	medical_history	calc	Does the infant have feeding problems
cardio_inst	medical_history	calc	Cardio vascular instability indicated?
cns_symp	medical_history	checkbox	Central Nervous System symptoms?
umbil	medical_history	radio	Umplical sepsis noted?
h2_startdate	medical_history	text	H2 blockers started?
curr_feed	medical_history	checkbox	Current feeding
feed_type	medical_history	checkbox	Type of feeding
abnorm_oth	medical_history	text	Other underlying abnormality, specify
antib_pos	medical_history	dropdown	Antimicrobial treatment at time of positive culture?
wcc_count	medical_history	text	White cell count (WCC) results
platelet_count	medical_history	text	Platelets

For peer review only - http://bmjopen.bmj.com/site/about/guidelines.xhtml

abg_res	medical_history	text	Arterial Blood Gas STANDARD BASE EXCESS (SBE in mmol/L or mEq/L)
ph_result	medical_history	text	What was the patients pH?
pco2_result	medical_history	text	What was the patients pCO2
co2_unit	medical_history	radio	What units is the pCO2 measured in
hc03_result	medical_history	text	What was the patients HCO3 (in mmol/L)
lumbar	medical_history	radio	Was lumbar puncture performed for positive culture?
csf_wcc	medical_history	text	CSF WCC results
csf_res	medical_history	radio	CSF Pleocytosis (for babies < 28 days and cell count >20cells/mm3; or babies >28 days cell count >10cells/mm3)
finout_inf	main_outcome	radio	Final outcome at end of hospitalisation or after 30 days of specimen collection if still admitted for other reasons
fin_date	main_outcome	text	Date of final outcome infant
neon_date	main_outcome	text	Date that NEONATE was 28 days old calculated from [birth_date] plus 27 days
outcome_neon	main_outcome	radio	Neonate Outcome at 28 days

For peer review only - http://bmjopen.bmj.com/site/about/guidelines.xhtml

BMJ Open

A study protocol for a population-based observational surveillance study of culture-confirmed neonatal bloodstream infections and meningitis in South Africa: Baby GERMS-SA

Journal:	*BMJ Open*
Manuscript ID	bmjopen-2021-049070.R1
Article Type:	Protocol
Date Submitted by the Author:	15-Dec-2021
Complete List of Authors:	Meiring, Susan; National Institute for Communicable Diseases; University of the Witwatersrand Faculty of Health Sciences<br>Mathebula, Rudzani; National Institute for Communicable Diseases<br>Magobo, Rindidzani; National Institute for Communicable Diseases<br>Perovic, Olga; National Institute for Communicable Diseases; University of the Witwatersrand Faculty of Health Sciences, Department of Clinical Microbiology and Infectious Diseases<br>Quan, Vanessa; National Institute for Communicable Diseases<br>Cohen, Cheryl; National Institute for Communicable Diseases<br>de Gouveia, Linda; National Institute for Communicable Diseases<br>von Gottberg, Anne ; National Institute for Communicable Diseases; University of the Witwatersrand Faculty of Health Sciences<br>Mackay, Cheryl; Dora Nginza Hospital, Department of Paediatrics and Child Health<br>Mailula, Mphekwa; Mankweng Regional Hospital Mankweng, Department of Paediatrics and Child Health<br>Phayane, Rose; Tembisa Provincial Hospital, Department of Paediatrics and Child Health<br>Dramowski, Angela; Stellenbosch University, Department of Paediactrics and Child Health, Division of Paediatric Infectious Diseases, Faculty of Medicine and Health Sciences<br>Govender, Nelesh; National Institute for Communicable Diseases; University of the Witwatersrand Faculty of Health Sciences
**Primary Subject Heading**:	Infectious diseases
Secondary Subject Heading:	Paediatrics
Keywords:	NEONATOLOGY, INFECTIOUS DISEASES, Paediatric infectious disease & immunisation < PAEDIATRICS, PUBLIC HEALTH, Microbiology < PATHOLOGY, Paediatric pathology < PAEDIATRICS

Title

A study protocol for a population-based observational surveillance study of culture-confirmed neonatal bloodstream infections and meningitis in South Africa: Baby GERMS-SA

Authors

Susan Meiring[1,2]; Rudzani Mathebula[3]; Rindidzani Magobo[3]; Olga Perovic[3,4]; Vanessa Quan[1]; Cheryl Cohen[2,5]; Linda de Gouveia[5]; Anne von Gottberg[4,5,6]; Cheryl Mackay[7]; Mphekwa T. Mailula[8]; Rose Phayane[9]; Angela Dramowski[10]; Nelesh P. Govender[3,4,6] for Baby GERMS-SA

Affiliations

1. Division of Public Health Surveillance and Response, National Institute for Communicable Diseases, a Division of the National Health Laboratory Service, Johannesburg, South Africa
2. School of Public Health, Faculty of Health Sciences, University of the Witwatersrand, Johannesburg, South Africa
3. Centre for Healthcare-Associated Infections, Antimicrobial Resistance and Mycoses, National Institute for Communicable Diseases, a Division of the National Health Laboratory Service, Johannesburg, South Africa
4. Department of Clinical Microbiology and Infectious Diseases, School of Pathology, Faculty of Health Sciences, University of Witwatersrand, Johannesburg, South Africa
5. Centre for Respiratory Diseases and Meningitis, National Institute for Communicable Diseases, a Division of the National Health Laboratory Service, Johannesburg, South Africa
6. Division of Medical Microbiology, School of Pathology, Faculty of Health Sciences, University of Cape Town, South Africa
7. Department of Paediatrics and Child Health, Dora Nginza Hospital, Nelson Mandela Bay, South Africa
8. Department of Paediatrics and Child Health, Mankweng Regional Hospital, Mankweng, South Africa

For peer review only - http://bmjopen.bmj.com/site/about/guidelines.xhtml

9. Department of Paediatrics and Child Health, Tembisa Provincial Hospital, Johannesburg, South Africa
10. Department of Paediatrics and Child Health, Division of Paediatric Infectious Diseases, Faculty of Medicine and Health Sciences, Stellenbosch University, Cape Town, South Africa

Corresponding author

Dr Susan Meiring

Address: C18 Microbiology Department, NHLS, Groote Schuur Hospital, Anzio Road, Observatory, 7925, South Africa.

Telephone: +27 21 404 5540 +27 84 597 3688

Email: susan.meiring@nhls.ac.za

Key words

Neonates; sepsis; bloodstream infection; meningitis; South Africa; surveillance; antimicrobial resistance

Word count:

Abstract: 249 (250)

Main body: 3272

Abstract

Introduction:

Worldwide, neonatal mortality remains high accounting for 47% of childhood deaths in 2019 and including an estimated 500 000 deaths from neonatal infections. While 42% of global neonatal deaths occur in Sub-Saharan Africa, there is limited understanding of population-level burden and aetiology of neonatal infections outside tertiary-level institutions.

Methods and analysis:

For peer review only

For peer review only - http://bmjopen.bmj.com/site/about/guidelines.xhtml

We aim to implement the first population-level surveillance for bloodstream infections and meningitis among neonates aged <28 days in South Africa. Tier 1 will include national surveillance of culture-confirmed neonatal infections at all public-sector hospitals describing infection incidence risk, pathogen profile and antimicrobial susceptibility by institution, province and healthcare level (2014-2021). Tier 2 (nested within tier 1) will be conducted at 6 regional neonatal units over 12 months, will compare the clinical characteristics of neonates with early- and late-onset infections and identify potentially-modifiable risk factors for mortality. Through tier 2, we will determine the antimicrobial susceptibility of neonatal pathogens, evaluate the appropriateness of empiric antibiotic prescribing and determine the genomic epidemiology of multi-drug resistant bacterial and fungal pathogens.

Ethics and dissemination:

Ethics clearance was obtained from the Human Research Ethics Committee of the University of the Witwatersrand (M190320). Funding for the study was obtained through a grant from the Bill and Melinda Gates Foundation (OPP1208882). Baby GERMS-SA aims to impact on national policy, resource allocation and neonatal guidelines by describing the national burden of neonatal infections in South Africa. In addition, end-users in neonatal units will benefit from a facility-level dashboard displaying key indicators of the surveillance findings.

Summary: Strengths and Limitations

- Baby GERMS-SA will be the first population-level surveillance study to determine the aetiology of culture-confirmed neonatal infections in an African country.
- Two complementary surveillance approaches will be used:
    - retrospective population-based laboratory surveillance in all public health facilities in South Africa
    - prospective enhanced surveillance for clinical data at six neonatal units
- Providing baseline incidence estimates for culture-confirmed early- and late-onset infections in neonatal units will help monitor the impact of future public health interventions aimed at reducing infection-related neonatal mortality.

For peer review only - http://bmjopen.bmj.com/site/about/guidelines.xhtml

For peer review only

- The observational nature of the study may underestimate neonatal infection burden in rural districts of South Africa, where specimen taking practices to confirm infections are sub-optimal

Introduction

Worldwide, neonatal mortality remains high, despite a substantial decline in under-5 childhood deaths from 12.7 million in 1990 to 5.2 million in 2019.[1][2] Neonatal deaths accounted for 47% of all under-5 childhood deaths in 2019, with infectious causes being the third highest contributors to neonatal mortality, following prematurity and intra-partum related events.[2] Infectious diseases caused approximately 500 000 neonatal and 1.5 million under-5 childhood deaths in 2017.[3][2]

While 42% of global neonatal deaths occur in Sub-Saharan Africa, the population-level burden and aetiology of neonatal infections is not well understood.[1,4,5] Studies in Africa have been limited to tertiary-level institutions, with no population-based surveillance studies reporting on neonatal infection incidence risks or rates.[5][6–8] Contributing factors to this lack of data include under-utilization or unavailability of hospital-based services for neonatal care, suboptimal specimen collection to confirm an infectious disease diagnosis, limited capacity of diagnostic pathology laboratories to detect, identify and characterize neonatal pathogens, absence of appropriate denominator data for calculating incidence risks or rates, lack of clinical data to differentiate between infection types (i.e. healthcare-associated infections versus vertical transmission of pathogens causing early-onset sepsis), and limited resources for setting up and maintaining population-based surveillance studies.[9–11]

The South African government seeks to reduce neonatal sepsis rates by 84% nationally by 2025 through various strategies across the continuum of maternal and newborn care.[12][13] However, unless the national burden of laboratory-confirmed neonatal infections occurring at all levels of health-care in South Africa can be clearly documented, measuring the effectiveness of these interventions against a baseline will be difficult.

We aim to improve the reporting of neonatal infection burden and determine the risk factors for mortality associated with neonatal infections in urban and rural South Africa

For peer review only - http://bmjopen.bmj.com/site/about/guidelines.xhtml

using a two-tiered surveillance study. We will describe the incidence of culture-confirmed neonatal bloodstream infections and meningitis by province, pathogen-specific aetiology and antimicrobial susceptibility at different levels of health-care over an eight-year period in tier 1. We will describe the clinical characteristics of culture-confirmed cases, identify modifiable risk factors associated with mortality and describe the antimicrobial susceptibility and genomic epidemiology of multi-drug resistant bacterial and fungal pathogens over 12 months in tier 2.

Methods

Hypothesis:

We hypothesise that the incidence risk of culture-confirmed neonatal infections has increased over the study period in South Africa, owing to in-hospital transmission of multi-drug-resistant organisms. In addition, we hypothesise that neonatal deaths due to infections may be related to modifiable risk factors such as low rates of antenatal steroid use in preterm infants, low rates of breastfeeding amongst neonates who develop infections, and prolonged use of indwelling catheters.[14,15]

Study objectives

Baby GERMS-SA has three main objectives:

i. To determine the bacterial and fungal aetiology and incidence risk of culture-confirmed infections amongst neonates presenting to all levels of hospital-based care in South Africa from 2014 to 2021

ii. To confirm the bacterial and fungal aetiology and prevalence of antimicrobial resistance in pathogens causing neonatal infections at secondary-level health-care facilities over a 12-month period in South Africa.

iii. To determine the characteristics of neonates who are diagnosed with culture-confirmed infections at secondary-level health care facilities and identify potentially-modifiable risk factors for death

For peer review only - http://bmjopen.bmj.com/site/about/guidelines.xhtml

Study design

Two complementary surveillance approaches will be used in this study. First, retrospective population-based surveillance will be established to identify culture-confirmed episodes of neonatal infections occurring in all public health facilities in South Africa from 2014 through to 2021, and population denominator data on live births will be used to calculate national and provincial incidence risks of infection (Tier 1). Second, prospective enhanced laboratory-based surveillance will be conducted at six sentinel neonatal units to collect detailed clinical data from neonates with infection and to determine risk factors for mortality (Tier 2). The overall and pathogen-specific incidence rate of neonatal infections will be calculated at sentinel sites using patient bed-days as a denominator. All-cause mortality rates will also be calculated. The sentinel neonatal units will be selected from a list of secondary level/ regional public-sector hospitals with inpatient neonatal services. Only one institution will be selected per province. In addition, a cross-sectional electronic survey will be conducted at a sample of large public-sector neonatal units in South Africa to determine available bed and staff resources, understand infection prevention and antimicrobial stewardship practices and obtain admission denominator data.

Definitions

A neonate will be defined as a child aged <28 days with further categorisation into early (0-6 days) and late neonatal periods (7-27 days). The post-neonatal period will be defined as the period from 28-60 days.

We will use a laboratory-based case definition for neonatal invasive infections based on Level 1 of the Brighton Collaboration Neonatal Infections Working Group for neonatal invasive bloodstream infections.[16] This includes any neonate/infant who is admitted to a public-sector hospital with a recognised pathogen (bacteria or fungi) identified using a validated method from a normally-sterile site (blood or cerebrospinal fluid [CSF]) or a normally non-pathogenic organism, e.g. coagulase-negative staphylococci isolated from 2 invasive specimen cultures taken at 2 different time points within 14-days. We will use a 14-day period from the date of the first positive culture to define an episode of infection. This case definition makes the assumptions that: i) the neonate/ infant would not have been evaluated for sepsis (i.e. had specimens collected for culture) in the absence of clinical signs

For peer review only - http://bmjopen.bmj.com/site/about/guidelines.xhtml

or a clear clinical indication and ii) the isolated bacteria/ fungi are not contaminants and iii) that most neonatal bacterial or fungal infections can be cleared within 14 days with appropriate antimicrobial treatment. We will use a 72-hour age cut off to distinguish early-onset (less than 72 hours since birth) from healthcare-associated (≥ 72 hours since birth) neonatal infections.

Study processes, Training and Analyses

Tier 1: National population-based laboratory-based surveillance

Positive blood and CSF microbiology culture results from patients admitted to public health institutions in South Africa will be obtained from a surveillance data warehouse which archives data from TrakCare, the electronic National Health Laboratory Service (NHLS) laboratory information system in use since at least 2014. We will request data on positive blood and CSF cultures among infants aged <12 months for at least an 8-year period (from 1 January 2014 through to 31 December 2021). Based on a preliminary analysis of 2016 data, we estimate 7 000-8 000 laboratory-confirmed neonatal infections to be reported each year from approximately 180 public-sector hospitals. (Figure 1) The following variables will be requested: laboratory name, province, district, sub-district, hospital name, ward name/ type, patient first name and surname, laboratory episode number, data warehouse unique identifier, patient date of birth, date of specimen collection, specimen type, microscopy (including Gram stain and CSF cell counts) and culture result, identification of pathogen and antimicrobial susceptibility results. Patient identifying information (i.e. name, date of birth) will be requested in order to accurately de-duplicate records; this is also the current practice in the "parent" GERMS-SA surveillance programme.[17] This information is essential to distinguish neonatal and maternal specimens (in the first few days of life, clinicians often send specimens labelled with the mother's details). Neonatal date of birth is also an essential piece of information to determine timing of infection (early versus late onset). A national surveillance dataset will be created containing de-duplicated laboratory records of neonates with laboratory-confirmed bloodstream and CSF infections. These data will be cleaned and analysed using Stata version 15 (StataCorp Inc., College Station, Texas, USA).

Data from the first tier of national laboratory-based surveillance will be used to calculate the incidence risk of neonatal sepsis stratified by level of healthcare (district (level 1), regional

For peer review only - http://bmjopen.bmj.com/site/about/guidelines.xhtml

(level 2) or tertiary/referral (level 3)), geographic region (province, district, sub-district) and timing of infection. We will use national neonatal unit admissions (if available through the cross-sectional survey) or live births in the total population as denominators for incidence risk calculations.[18] The main analysis (incidence risk calculations) may focus on provinces where specimen-collection practices are more consistent and estimates of incidence risks are more likely to be valid. Missing data will be imputed for the stratified incidence risk calculations. In line with NICD's mandate, we will endeavour to make aggregate data publicly available through a neonatal infection dashboard displaying interactive maps and graphs to district level (similar to the antimicrobial resistance dashboard available at www.nicd.ac.za).

Tier 2: Enhanced sentinel site laboratory-confirmed neonatal sepsis surveillance

The six sentinel regional hospitals and their provinces include: Dora Nginza Provincial Hospital (Eastern Cape Province), Tembisa Hospital (Gauteng Province), Mankweng Hospital (Limpopo Province), Klerksdorp (North West Province), Queen Nandi Regional Hospital (KwaZulu-Natal Province) and Rob Ferreira Hospital (Mpumalanga Province) (Figure 1). In 2016, thirteen per cent (901/7 124) of all culture-confirmed neonatal infection episodes in South Africa occurred in neonates admitted to these facilities. (Figure 1) NHLS microbiology laboratories serving each of the six sentinel surveillance sites will be requested to prospectively submit any cultured bloodstream or CSF isolate from infants aged <12 months on Dorset transport media (Media Mage, Johannesburg) to the NICD reference laboratories for further characterisation. NICD personnel will provide training to participating laboratories to ensure that neonatal specimens are optimally processed and quality control measures are adhered to. In addition, training on diagnosis of neonatal sepsis and meningitis will be provided to clinicians. Data on basic demographic details of the infants and NHLS laboratory characterisation of the isolates will be transferred from the data warehouse directly into REDCap, an electronic data capture tool hosted at the University of the Witwatersrand, and reference laboratory isolate characterisation and clinical data from the infants will be added to this dataset. [19,20] We will prospectively monitor cases of neonatal infections at the sentinel hospitals and upon request, conduct investigations for any potential clusters/ outbreaks. These activities are covered by a separate ethics application (WITS HREC reference: M160667 – Essential communicable diseases surveillance

For peer review only - http://bmjopen.bmj.com/site/about/guidelines.xhtml

and outbreak investigation activities of the NICD). For each episode of culture-confirmed infection, we will perform a retrospective medical record review using a standardised data abstraction tool (supplementary table). Inpatient medical charts will be scanned after patient identifiers are physically blocked by a card with a unique study identifier. These imaged records will be abstracted electronically off-site by trained medical and nursing study personnel into a REDCap database.

A dataset will be generated containing all cases of neonatal infection from the six sentinel hospitals. This will include demographic and baseline clinical details of enrolled participants, clinical updates during their hospital admission, antimicrobial therapy during the admission, in-hospital outcome and detailed laboratory characterisation of the isolate/s causing infection. The following variables will be collected: i) Isolate data: laboratory name, province, district, sub-district, hospital name, ward name/ type, patient name and surname, laboratory episode number, data warehouse unique identifier, date of birth, date of admission, date of specimen collection, specimen type, microscopy and culture result, antimicrobial susceptibility results, and NICD reference laboratory data including whole genome sequencing (WGS) where applicable; ii) Additional patient data: basic demographic data, type of admitting unit, date of birth, gestational age at birth, mode of delivery, 5-min Apgar score, date of admission, comorbid neonatal conditions, maternal and neonatal risk factors for infection, maternal HIV status (and baby's HIV-PCR result if tested), presence of central venous lines, respiratory support (non-invasive, invasive, surfactant administration), clinical presentation on day of specimen collection, inflammatory markers of infection on day of specimen collection, empiric and directed antibiotic/antifungal therapy (determine if "appropriate" based on organism susceptibility), complications of infection, in-hospital outcome at 28 days (for neonates) or at the end of admission (for neonates and infants who are admitted for longer periods).

The following analyses will be performed using this dataset: i) A description of the characteristics of neonates with culture-confirmed bloodstream infection and meningitis by pathogen; ii) A description of the antimicrobial susceptibility of the most important bacterial and fungal pathogens causing neonatal infections in South Africa over time and appropriateness of current empiric regimens for early and late-onset infections iii) The incidence risk of infection by pathogen per 1 000 live births in the catchment area or per 1

For peer review only - http://bmjopen.bmj.com/site/about/guidelines.xhtml

000 inpatient days will be calculated for neonates (0-27 days) iv) An estimation of the outcomes following neonatal infection and the risk factors associated with death v) Multivariable analyses will be performed to determine potentially-modifiable risk factors for mortality for various subgroups (e.g. effect of receiving antenatal steroids on mortality amongst preterm infants; effect of feeding mode on mortality; effect of prolonged use of indwelling catheters on mortality among those with late-onset healthcare-associated infections). For each analysis, we will adjust for potential confounders (such as sex, birthweight, preterm birth) as appropriate.

As this dataset will contain confidential information whereby individuals could potentially be identified, the complete dataset will not be available publically. However, should external researchers request any of this information, completely de-identified data may be released following signing of a data-sharing agreement between the NICD and the requestor.

Electronic survey of neonatal units with previously-identified episodes of neonatal sepsis

A baseline survey of neonatal units will be conducted in 2020 to improve our understanding of the current functioning of neonatal units at various health-care levels in South Africa. A standardised questionnaire will be sent to all dedicated in-patient neonatal units for the facility manager to complete in hard copy, electronically or online using the SurveyMonkey application. Of 7 124 episodes of laboratory-confirmed neonatal infections diagnosed at 179 South African public hospitals in 2016, 97% (6 919 episodes) were from 90 hospitals that had >5 episodes of infection. We will approach these 90 hospitals for the neonatal unit survey. (Figure 1) Variables to be collected will include: hospital location, bed census in the neonatal unit, number of neonatal unit staff members, on-site or off-site laboratory services, infection prevention and control (IPC) and clinical microbiologist support for the unit and detailed statistics on number of patient-days per month and deaths per month from the neonatal unit in 2019/2020. Clinical criteria used for suspicion of sepsis or bloodstream infections, criteria for obtaining blood/CSF specimens for culture from neonates and institutional antibiotic guidance for early- and late-onset sepsis will be also be collected. Completed questionnaire data will be captured into Microsoft Excel and analysis performed using Stata. We will use denominator data obtained through the survey (e.g. admissions, patient-days) for incidence risk/ rate calculations at tier 2 facilities.

For peer review only - http://bmjopen.bmj.com/site/about/guidelines.xhtml

Data dissemination

The information gained through the surveillance study will be shared with the South African Department of Health, and various other in-country and international stakeholders. Internally, the data will be presented to the National Advisory Group on Immunisations, the Ministerial Advisory Committee on Antimicrobial Resistance, Neonatal Sepsis Task Force and the multi-sectoral National Outbreak Response Team, as well as at local and international conferences.[21] We plan to publish the results in a policy-briefing and in relevant peer-reviewed medical journals. A facility-level dashboard used to display key indicators based on the surveillance data will be set up and made available to end-users in neonatal units.

Beneficiaries

We will gain a better understanding of the burden of neonatal infections and the antimicrobial susceptibility and molecular relatedness of neonatal bacterial and fungal pathogens in an upper middle-income country, particularly in secondary-level institutions serving peri-urban and rural communities. The National Department of Health could use these data to design appropriate interventions such as antimicrobial stewardship and infection prevention and control programmes, to prioritise facilities requiring urgent intervention and to tailor these interventions for those at highest risk of neonatal sepsis. The hospitals at which we will conduct enhanced surveillance will benefit from the additional information that will come from further characterization of the isolates causing neonatal infections and gain an understanding on how they can tailor their empiric antimicrobial regimens to fit the spectrum of organisms that are being cultured. Individual neonates at these hospitals will thus benefit from the doctors adjusting their empirical therapy accordingly. We will strongly encourage enhanced surveillance sites to implement local antimicrobial stewardship and infection prevention and control programs for neonatal units and will design facility-level reports based on their local surveillance data to allow them to monitor key indicators for neonatal sepsis. Policy makers can use the data on burden of disease, mortality and risk factors associated with neonatal sepsis and the aetiological patterns of pathogens causing neonatal sepsis to align their strategies on the Continuum of Maternal and Newborn Care to help meet South Africa's goal to reduce neonatal sepsis by 84% by the year 2025. We hope that by setting up this surveillance study,

For peer review only - http://bmjopen.bmj.com/site/about/guidelines.xhtml

we will facilitate future sustainable funding of the project and will be able to objectively record the change in incidence risk of neonatal infections over time as new interventions are implemented. Ultimately, we hope that policies put in place through the data generated by this project will save the lives of many newborn babies and improve the quality of life of others in the years ahead.

Patient and public involvement

The study design and protocol was discussed with paediatricians and neonatologists at various institutions across South Africa, as well as at the launch of the Neonatal Sepsis Task Force at the United SA Neonatal Association (USANA) conference in Port Elizabeth, SA, on 13 September 2019.

Ethics and funding

The study has been approved by the Human Research Ethics Committee of the University of the Witwatersrand (M190320). Approvals for the tier 2 surveillance study were received from each provincial research committee through registration on the National Health Research Database. Funding for the study was awarded as a grant to N.P.G. from the Bill and Melinda Gates Foundation (OPP1208882).

Discussion

Unless baseline data on neonatal infections are reliably and systematically collected and analysed, there will be no objective record against which interventions aimed at reducing burden of disease in this vulnerable population can be measured. The Baby GERMS-SA national surveillance system will provide a robust platform to determine national incidence risk of neonatal infection by pathogen, level of hospital care and geographic region. This surveillance study will also be used to assess trends in the incidence risk of neonatal infections over time, thus providing an objective record by which to measure the impact of any intervention implemented in future.

The enhanced surveillance tier will focus on neonatal sepsis and meningitis occurring at sentinel hospitals. This surveillance tier will provide insight into the similarities/differences

For peer review only - http://bmjopen.bmj.com/site/about/guidelines.xhtml

in pathogens and their antimicrobial susceptibility patterns causing neonatal sepsis by level of health care, and risk factors for mortality among neonates admitted to regional hospitals. The NICD has experience in conducting surveillance for laboratory-confirmed meningitis as well as active surveillance of several invasive bacterial and fungal diseases at a national level.[22,23] We have previously published data indicating how public health interventions such as new vaccines, antiretroviral treatment, cryptococcal antigen screening and treatment have reduced the burden of disease/ mortality caused by *Streptococcus pneumoniae, Haemophilus influenzae* and *Cryptococcus neoformans* in South Africa.[24,25][26] We realise the value of having robust data prior to implementation of public health interventions in reporting the effectiveness of these interventions over time. We also conduct surveillance on selected healthcare-associated bloodstream infections and understand the variation of antimicrobial resistance and pathogen profiles by healthcare level and province.[27] We are aware that the study findings rely on adequate specimen collection and laboratory diagnostic capacity of each neonatal unit. Specimen taking practices might differ between units and therefore the surveillance may underestimate neonatal infection burden, especially in rural districts of South Africa. Tier one's comprehensive dataset should be able to provide an expected culture-positivity rate to describe the extent of this phenomenon. A major strength of our project is that we will gather complete data on laboratory-confirmed bloodstream infections and meningitis from the entire public-sector population and in-depth data from selected secondary-level sites in six provinces.

Ultimately, these surveillance data can be used to address Sustainable Development Goal 3 by aiming to improve neonatal and child health by using a two-tiered laboratory-based surveillance program to gain a deeper understanding of the aetiology and burden of neonatal sepsis, with a future aim of addressing these factors and thus reducing neonatal morbidity and mortality in low- and middle-income settings.

Acknowledgements

The authors would like to acknowledge the following clinical and laboratory staff involved in the protocol design, facilitation and collection of data for the Baby GERMS-SA project: Dora

For peer review only - http://bmjopen.bmj.com/site/about/guidelines.xhtml

Nginza Hospital: Phunyezwa Mzayiya (laboratory), Shareef Abrahams (pathologist), Vanessa Pearce (laboratory), Zikhona Gabazana (research assistant (RA)), Melissa Ngubane (RA), Badikazi Matiwana (RA); Klerksdorp Hospital: Omphile Mekgoe (clinician), Sebabatso Khantsi (laboratory), Bernard Motsetse (RA), LouisaPhalatse (RA); Mankweng Hospital: Ruth Lekalakala (pathologist), Tebogo Modiba (RA), Molly Morapeli (RA); National Institute for Communicable Diseases: Linda Erasmus (pathologist), Danie Erwee (clinician), Juliet Paxton (clinician), Siyanda Dlamini (laboratory), Marshagne Smith (laboratory), Ruth Mpembe (laboratory), Ntombi Dube (administrator), Relebohile Ramatsa (RA), Thembekile Zwane (masters student), Sibongile Walaza (medical epidemiologist), Erika van Schalkwyk (medical epidemiologist); Queen Nandi Hospital: ; Constance Kapongo (clinician), Meluleki Mthimkhulu (laboratory), Sandra Maphumulo (pathologist), Dianette Pearce (RA); Rob Ferreira Hospital: Lerato Motjale (clinician), Thulisile Maphosa (clinician), Greta Hoyland (laboratory), Sindile Ntuli (pathologist), Lesley Ingle (RA); Tembisa Hospital: Harishia Naidoo (clinician), Ramatlhwa Kekana (laboratory), Dina Pombo (laboratory).

The authors would like to acknowledge Jimmy Khosa from the NICD for the production of the ARCGIS map for figure 1.

Funding

Funding for the study was awarded as a grant to N.P.G. from the Bill and Melinda Gates Foundation (OPP1208882).

Conflict of interest

The authors have no conflicts of interest to declare.

Contributorship statement

The authors contributed in the following ways:

- Substantial contribution to conception and design of the study: Susan Meiring, Rudzani Mathebula, Rindidzani Magobo, Olga Perovic, Vanessa Quan, Cheryl Cohen, Linda de Gouveia, Anne von Gottberg, Cheryl Mackay, Mphekwa T. Mailula, Rose Phayane, Angela Dramowski, Nelesh P. Govender

For peer review only - http://bmjopen.bmj.com/site/about/guidelines.xhtml

- Revising the manuscript for important intellectual content: Susan Meiring, Rudzani Mathebula, Nelesh P. Govender, Angela Dramowski, Vanessa Quan, Olga Perovic
- Final approval of manuscript: Susan Meiring, Rudzani Mathebula, Rindidzani Magobo, Olga Perovic, Vanessa Quan, Cheryl Cohen, Linda de Gouveia, Anne von Gottberg, Cheryl Mackay, Mphekwa T. Mailula, Rose Phayane, Angela Dramowski, Nelesh P. Govender
- Guarantor: Susan Meiring is the guarantor of this publication and takes full responsibility for the content, the decision to publish and the completed work.

Data statement

A facility-level dashboard used to display key indicators based on the surveillance data will be set up and made available to end-users at neonatal units. As the tier 2 dataset will contain confidential information whereby individuals could potentially be identified, the clinical dataset will not be available publically. However, should other researchers request any of this information, completely de-identified data may be released following signing of a data-sharing agreement between the NICD and the requestor.

Figures

Figure 1: Map of South Africa showing relative numbers of laboratory-confirmed bacterial and fungal infectious episodes amongst neonates diagnosed at each hospital site, 2016 (n=7 124)

*the six tier 2 sentinel surveillance sites are indicated in green

References

1. Sharrow D, Hug L, Liu Y, You D, United Nations Inter-agency Group for Child Mortality Estimation UNIG for CME. UN IGME Child mortality report 2019. 2019: 52. Available at: https://www.unicef.org/media/60561/file/UN-IGME-child-mortality-report-2019.pdf.

2. Liu L, Johnson H, Cousens S. Global, regional, and national causes of child mortality in 2000-2010: an updated systematic analysis. Lancet **2015**; 385:430–440.

For peer review only - http://bmjopen.bmj.com/site/about/guidelines.xhtml

3. Dicker D, Nguyen G, Abate D, et al. Global, regional, and national age-sex-specific mortality for 282 causes of death in 195 countries and territories, 1980-2017: a systematic analysis for the Global Burden of Disease Study 2017 GBD 2017 Causes of Death Collaborators*. Lancet **2018**; 392:1736–88.

4. Seale AC, Mwaniki M, Newton CR, Berkley JA. Maternal and early onset neonatal bacterial sepsis: burden and strategies for prevention in sub-Saharan Africa. Lancet Infect Dis **2009**; 9:428–438.

5. Fleischmann-Struzek C, Goldfarb DM, Schlattmann P, Schlapbach LJ, Reinhart K, Kissoon N. The global burden of paediatric and neonatal sepsis: a systematic review. Lancet Respir Med **2018**; 6:223–230.

6. Ogunlesi TA, Ogunfowora OB, Osinupebi O, Olanrewaju DM. Changing trends in newborn sepsis in Sagamu, Nigeria: bacterial aetiology, risk factors and antibiotic susceptibility. J Paediatr Child Heal **2011**; 47:5–11.

7. Hamer DH, Darmstadt GL, Carlin JB, et al. Etiology of bacteremia in young infants in six countries. Pediatr Infect Dis J **2015**; 34:e1-8.

8. Shitaye D, Asrat D, Woldeamanuel Y, Worku B. Risk factors and etiology of neonatal sepsis in Tikur Anbessa University Hospital, Ethiopia. Ethiop Med J **2010**; 48:11–21.

9. Lawn JE, Bianchi-Jassir F, Russell NJ, et al. Group B Streptococcal Disease Worldwide for Pregnant Women, Stillbirths, and Children: Why, What, and How to Undertake Estimates? Clin Infect Dis **2017**; 65:S89–S99.

10. The World Health Organisation, UNICEF. Managing possible serious bacterial infection in young infants when referral is not feasible: Guidelines and WHO/UNICEF recommendations for implementation. 2015. Available at: http://www.who.int/maternal_child_adolescent/documents/bacterial-infection-infants/en/. Accessed 7 December 2021.

11. Williams PCM, Isaacs D, Berkley JA. Antimicrobial resistance among children in sub-Saharan Africa. Lancet Infect Dis **2018**; 18:e33–e44.

12. Lawn JE, Kinney M, Blencowe H, Group The Lancet Every Newborn Study. Every

For peer review only

For peer review only - http://bmjopen.bmj.com/site/about/guidelines.xhtml

Newborn: Executive summary. Lancet **2014**; Every newb:1–8.

13. Kerber KJ, de Graft-Johnson JE, Bhutta ZA, Okong P, Starrs A, Lawn JE. Continuum of care for maternal, newborn, and child health: from slogan to service delivery. Lancet **2007**; 370:1358–1369.

14. Mwansa-Kambafwile J, Cousens S, Hansen T, Lawn JE. Antenatal steroids in preterm labour for the prevention of neonatal deaths due to complications of preterm birth. Int J Epidemiol **2010**; 39.

15. Griffin JB, Jobe AH, Rouse D, McClure EM, Goldenberg RL, Kamath-Rayne BD. Evaluating WHO-recommended interventions for preterm birth: A mathematical model of the potential reduction of preterm mortality in sub-Saharan Africa. Glob Heal Sci Pract **2019**; 7:215–227.

16. Vergnano S, Buttery J, Cailes B, et al. Neonatal infections: Case definition and guidelines for data collection, analysis, and presentation of immunisation safety data. Vaccine **2016**; 34:6038–6046.

17. Group for Enteric Respiratory and Meningeal disease Surveillance in South Africa. GERMS-SA Annual Report 2008. 2009. Available at: https://www.nicd.ac.za/assets/files/2008_GERMS-SA_Annual_Report(1).pdf. Accessed 7 December 2021.

18. Statistics South Africa . Statistical Release: Recorded Live Births: South Africa 2019. 2019. Available at: https://www.statssa.gov.za/publications/P0305/P03052019.pdf. Accessed 7 December 2021.

19. Harris PA, Taylor R, Thielke R, Payne J, Gonzalez N, Conde JG. Research electronic data capture (REDCap)-A metadata-driven methodology and workflow process for providing translational research informatics support. J Biomed Inform **2009**; 42:377–381.

20. Harris PA, Taylor R, Minor BL, et al. The REDCap consortium: Building an international community of software platform partners. J Biomed Inform **2019**; 95.

21. Dramowski A, Velaphi S, Reubenson G, et al. National Neonatal Sepsis Task Force

For peer review only - http://bmjopen.bmj.com/site/about/guidelines.xhtml

launch: Supporting infection prevention and surveillance, outbreak investigation and antimicrobial stewardship in neonatal units in South Africa. South African Med J **2020**; 110:360–363.

22. GERMS-SA. GERMS-SA Annual Report, 2018. 2019. Available at: https://www.nicd.ac.za/wp-content/uploads/2019/11/GERMS-SA-AR-2018-Final.pdf. Accessed 7 December 2021.

23. Britz E, Perovic O, von Mollendorf C, et al. The Epidemiology of Meningitis among Adults in a South African Province with a High HIV Prevalence, 2009-2012. PLoS One **2016**; 11:e0163036.

24. von Gottberg A, de Gouveia L, Madhi SA, et al. Impact of conjugate Heamophilus influenzae type b (Hib) vaccine introduction in South Africa. Bull World Heal Organ **2006**; 84:811–818.

25. von Gottberg A, de Gouveia L, Tempia S, et al. Effects of vaccination on invasive pneumococcal disease in South Africa. N Engl J Med **2014**; 371:1889–1899.

26. Larson BA, Rockers PC, Bonawitz R, et al. Screening HIV-Infected patients with low cd4 counts for cryptococcal antigenemia prior to initiation of antiretroviral therapy: Cost effectiveness of alternative screening strategies in South Africa. PLoS One **2016**; 11.

27. Perovic O, Iyaloo S, Kularatne R, et al. Prevalence and Trends of Staphylococcus aureus Bacteraemia in Hospitalized Patients in South Africa, 2010 to 2012: Laboratory-Based Surveillance Mapping of Antimicrobial Resistance and Molecular Epidemiology. PLoS One **2015**; 10:e0145429.

For peer review only

For peer review only - http://bmjopen.bmj.com/site/about/guidelines.xhtml

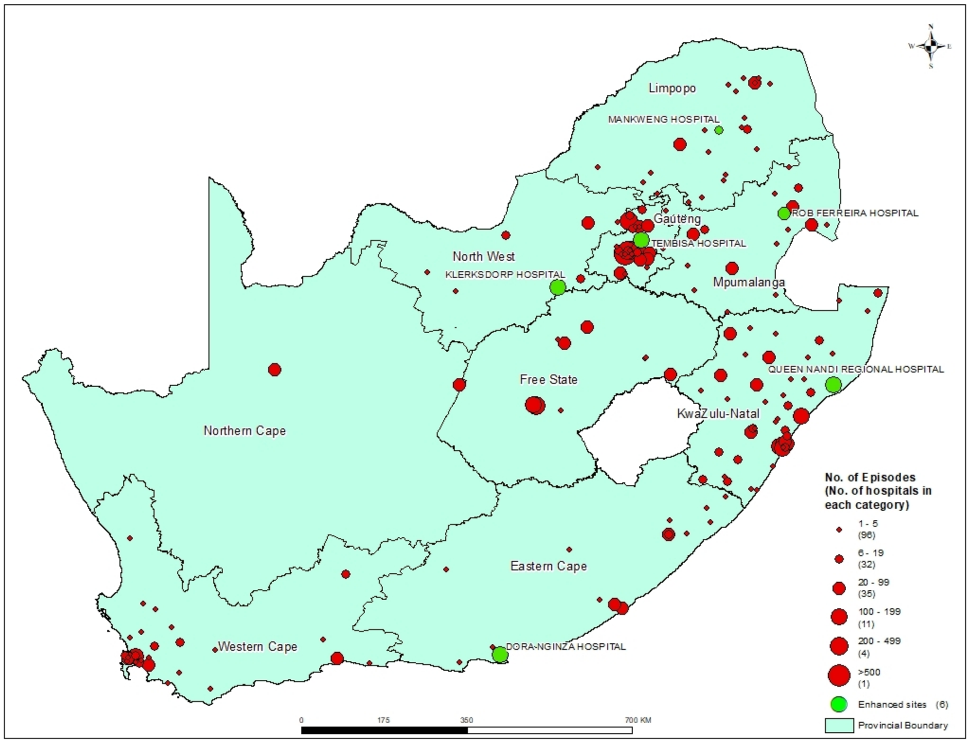

Figure 1: Map of South Africa showing relative numbers of laboratory-confirmed bacterial and fungal infectious episodes amongst neonates diagnosed at each hospital site, 2016 (n=7 124)

279x215mm (96 x 96 DPI)

A study protocol for a population-based observational surveillance study describing culture–confirmed neonatal bloodstream infections and meningitis in South Africa: Baby GERMS-SA

Authors

Susan Meiring[1,2]; Rudzani Mathebula[3]; Rindidzani Magobo[3]; Olga Perovic[3,4]; Vanessa Quan[1]; Cheryl Cohen[2,5]; Linda de Gouveia[5]; Anne von Gottberg[4,5,6]; Cheryl Mackay[7]; Mphekwa T. Mailula[8]; Rose Phayane[9]; Angela Dramowski[10]; Nelesh P. Govender[3,4,6] for Baby GERMS-SA

Affiliations

1. Division of Public Health Surveillance and Response, National Institute for Communicable Diseases, a Division of the National Health Laboratory Service, Johannesburg, South Africa
2. School of Public Health, Faculty of Health Sciences, University of the Witwatersrand, Johannesburg, South Africa
3. Centre for Healthcare-Associated Infections, Antimicrobial Resistance and Mycoses, National Institute for Communicable Diseases, a Division of the National Health Laboratory Service, Johannesburg, South Africa
4. Department of Clinical Microbiology and Infectious Diseases, School of Pathology, Faculty of Health Sciences, University of Witwatersrand, Johannesburg, South Africa
5. Centre for Respiratory Diseases and Meningitis, National Institute for Communicable Diseases, a Division of the National Health Laboratory Service, Johannesburg, South Africa
6. Division of Medical Microbiology, School of Pathology, Faculty of Health Sciences, University of Cape Town, South Africa
7. Department of Paediatrics and Child Health, Dora Nginza Hospital, Nelson Mandela Bay, South Africa
8. Department of Paediatrics and Child Health, Mankweng Regional Hospital, Mankweng, South Africa
9. Department of Paediatrics and Child Health, Tembisa Provincial Hospital, Johannesburg, South Africa
10. Department of Paediatrics and Child Health, Division of Paediatric Infectious Diseases, Faculty of Medicine and Health Sciences, Stellenbosch University, Cape Town, South Africa

For peer review only

For peer review only - http://bmjopen.bmj.com/site/about/guidelines.xhtml

Supplementary table: Baby GERMS neonatal sepsis surveillance project data dictionary for tier 2 enhanced surveillance data collection tool on REDCap

Variable / Field Name	Form Name	Field Type	Field Label
study_id	lab_form	text	Patient Study ID
episode_no	lab_form	text	Episode Number
patient_surname	lab_form	text	Patient Surname
patient_name	lab_form	text	Patient First Names
birth_date	lab_form	text	Date of birth
gender	lab_form	text	Gender
folder_no	lab_form	text	Folder Number
spec_date	lab_form	text	First specimen collection date
hosp_name	lab_form	text	Hospital name
province_name	lab_form	text	Province name
spec_type	lab_form	text	Specimen type
organism_name	lab_form	text	Organism Name
received_by_charm	lab_form	text	Received by CHARM
specm_date	isolates	text	Specimen Collection date
nicu_admdate	treatment_history	text	NICU admission date
pt_ref	treatment_history	dropdown	Was patient referred from another hospital
ref_date	treatment_history	text	Date of admission at [ref_facility]
ref_facility	treatment_history	text	If Yes, state facility name where patient was referred from
ref_days	treatment_history	calc	Number of days at referred facility [ref_facility] before transferred to [hosp_name]
adm_birth_yes	treatment_history	radio	Was the baby admitted since birth [yes/no]
trans_outfac	treatment_history	dropdown	Was patient transferred to another hospital?
date_transf	treatment_history	text	Date transferred out to [fac_name]
fac_name	treatment_history	text	If Yes, specify hospital name
momhiv_status	treatment_history	radio	Maternal HIV Status of [mom_surname] [mom_name]
hivstatus_child	treatment_history	radio	HIV status child
hivtest	treatment_history	radio	HIV test type
hiv_date	treatment_history	text	HIV Test date
child_exposure	treatment_history	radio	Was the child exposed to HIV-infection [exposed/unexposed]

For peer review only - http://bmjopen.bmj.com/site/about/guidelines.xhtml

antib_prior	treatment_history	dropdown	Antimicrobial treatment in 2 weeks prior to culture?
diagn_organism	treatment_history	checkbox	Diagnosis related to organism isolated
del_mode	antenatal_history	dropdown	Type of delivery
gestage	antenatal_history	text	Gestational age at birth
prem_reason	antenatal_history	radio	If Gestational age of [gestage] weeks is below 37, state premature reasons
mat_cond	antenatal_history	checkbox	Maternal condition
fet_cond	antenatal_history	checkbox	Fetal conditions
antib_intrap	antenatal_history	dropdown	Were intrapartum antibiotics given to the mother?
antib_intra	antenatal_history	checkbox	If YES state antibiotics prescribed and dates
birth_wght	antenatal_history	text	Birth weight
pregn_type	antenatal_history	dropdown	Pregnancy type [singleton, twin, triplet, etc]
birth_plc	antenatal_history	dropdown	Place of birth
home_birth	antenatal_history	dropdown	If delivery at Home, was birth attended?
resusc	antenatal_history	dropdown	Was resuscitation required at birth?
resusc_type	antenatal_history	radio	Types of resuscitation
resusc_oth	antenatal_history	text	If Other resuscitation, specify
apgr_1min	antenatal_history	text	Apgar score at 1 minute
apgr_5min	antenatal_history	text	Apgar score at 5 minutes
apgr_10min	antenatal_history	text	Apgar score at 10 minutes
anten_steroids	antenatal_history	dropdown	Antenatal steroids use
preg_num	antenatal_history	text	Total number of pregnancies (gravidity)
preg_birth	antenatal_history	text	Total number of births (parity) include this child in the number
mom_age	antenatal_history	text	Maternal age of [mom_surname] [mom_name] in Years
mom_educ	antenatal_history	dropdown	Maternal education of [mom_surname] [mom_name]
relat_status	antenatal_history	dropdown	Relationship status of [mom_surname] [mom_name]
empl_status	antenatal_history	dropdown	Employment status of Mother, [mom_name] [mom_surname]
preg_compl	antenatal_history	radio	Any pregnancy related infection?

For peer review only - http://bmjopen.bmj.com/site/about/guidelines.xhtml
For peer review only

curr_wght	medical_history	text	Current weight done closest to specimen date [spec_date]
invasive	medical_history	radio	Invasive devices required by the child?
invas_spec	medical_history	checkbox	If Invasive = [invasive] specify, select all that apply
pulse_rate	medical_history	radio	Pulse rate
resp_rate	medical_history	text	Respiratory rate
tacchyp_yes	medical_history	radio	Tacchypnea present?
tacchyp_no	medical_history	radio	Tacchypnea present?
oxyg_res	medical_history	text	Oxygen saturation
bld_syst	medical_history	text	Blood pressure result (Systolic)
bld_diast	medical_history	text	Blood pressure result (Diastolic)
temp_rest	medical_history	text	Temperature result
resp_dist	medical_history	calc	Respiratory distress symptoms?, (Yes, if any of options 1-7 is checked
resp_req	medical_history	calc	Respiratory support required? (Yes, if any of options 1-6 is checked, No, if None is checked)
resp_improv	medical_history	radio	Has respiratory support increased since day prior to specimen collection? Ie increased ventilation, new intubation, more O2 required
feed_prob	medical_history	calc	Does the infant have feeding problems
cardio_inst	medical_history	calc	Cardio vascular instability indicated?
cns_symp	medical_history	checkbox	Central Nervous System symptoms?
umbil	medical_history	radio	Umplical sepsis noted?
h2_startdate	medical_history	text	H2 blockers started?
curr_feed	medical_history	checkbox	Current feeding
feed_type	medical_history	checkbox	Type of feeding
abnorm_oth	medical_history	text	Other underlying abnormality, specify
antib_pos	medical_history	dropdown	Antimicrobial treatment at time of positive culture?
wcc_count	medical_history	text	White cell count (WCC) results
platelet_count	medical_history	text	Platelets
abg_res	medical_history	text	Arterial Blood Gas STANDARD BASE EXCESS (SBE in mmol/L or mEq/L)

For peer review only - http://bmjopen.bmj.com/site/about/guidelines.xhtml

For peer review only

ph_result	medical_history	text	What was the patients pH?
pco2_result	medical_history	text	What was the patients pCO2
co2_unit	medical_history	radio	What units is the pCO2 measured in
hc03_result	medical_history	text	What was the patients HCO3 (in mmol/L)
lumbar	medical_history	radio	Was lumbar puncture performed for positive culture?
csf_wcc	medical_history	text	CSF WCC results
csf_res	medical_history	radio	CSF Pleocytosis (for babies < 28 days and cell count >20cells/mm3; or babies >28 days cell count >10cells/mm3)
finout_inf	main_outcome	radio	Final outcome at end of hospitalisation or after 30 days of specimen collection if still admitted for other reasons
fin_date	main_outcome	text	Date of final outcome infant
neon_date	main_outcome	text	Date that NEONATE was 28 days old calculated from [birth_date] plus 27 days
outcome_neon	main_outcome	radio	Neonate Outcome at 28 days

For peer review only

For peer review only - http://bmjopen.bmj.com/site/about/guidelines.xhtml
